# USleep: efficacy of app-based audio interventions to improve sleep disturbance in working adults, a multi-arm randomized controlled trial

**DOI:** 10.1093/sleep/zsag154

**Published:** 2026-06-01

**Authors:** Jessica Vazzaz, Faith Matcham, Marcos Economides, Heather Bolton, Kate Cavanagh

**Affiliations:** School of Psychology, University of Sussex, Falmer, United Kingdom; School of Psychology, University of Sussex, Falmer, United Kingdom; Science Department, Unmind Ltd, London, United Kingdom; Science Department, Unmind Ltd, London, United Kingdom; School of Psychology, University of Sussex, Falmer, United Kingdom

**Keywords:** sleep, digital intervention, randomized controlled trial, smartphone apps, efficacy

## Abstract

**Study objectives:**

To evaluate the efficacy of three categories of standalone, audio-based sleep interventions (Bedtime Stories, Sleep Sounds, Sleep Skills) delivered via mental health application (MHapp) in improving sleep among working adults with sleep disturbance.

**Methods:**

A multi-arm, parallel randomized controlled trial was conducted. Adults with self-reported sleep disturbances were recruited online and randomly allocated to Bedtime Stories, Sleep Sounds, Sleep Skills, or digital control. Participants completed self-report questionnaires on sleep disturbance and other related outcomes at baseline (t0) and after the 4-week intervention (t1). The primary analysis followed an intention-to-treat approach using mixed-effects models.

**Results:**

A total of 495 working adults (mean age = 32.7 years; 55.8% female) were randomized. For sleep disturbance (primary outcome), the between-group Hedges’ *g* effect sizes were very small and not statistically significant (Bedtimes stories vs. control: *g* = 0.12, 95% CI −0.13 to 0.37, Sleep Sounds vs. control: *g* = 0.14, 95% CI −0.11 to 0.39, Sleep Skills 0.07, 95% CI −0.07 to 0.29), with slightly greater reductions in sleep disturbance for the intervention groups than control. The same pattern was observed for sleep-related impairment, mental health, well-being, and pre-sleep arousal.

**Conclusion:**

Audio-based sleep interventions delivered via a MHapp did not demonstrate superior efficacy over a digital control condition in reducing self-reported sleep disturbance among working adults. Although safe and well-tolerated, their use as standalone treatments for sleep disturbance is not supported by these findings. Future research should explore effectiveness in real-world settings, including user content choice across categories, and use objective sleep measures.

**Clinical Trial Registration:**

Registered at https://www.isrctn.com/ under “Evaluating the efficacy of audio-based digital tools to improve sleep on the Unmind workplace well-being platform”; https://www.isrctn.com/ISRCTN13426045; registration number: 13426045.

## Teaser

Sleep disturbance affects a substantial proportion of the general population and has significant societal consequences. The current study aimed to evaluate the effectiveness of standalone, audio-based interventions for improving sleep in working adults, as these tools are popular and could offer scalable solutions. To date, this is the first study to assess their efficacy as a standalone bedtime intervention against a digital control condition. The findings suggest that, although these interventions are safe, acceptable, feasible, and well-received, they do not appear to confer greater benefits than brief, non-sleep–focused daytime engagement with a digital control condition. These results inform considerations for users and clinicians, as well as for researchers in sleep and digital health.

## Graphical Abstract

**Graphical Abstract:**
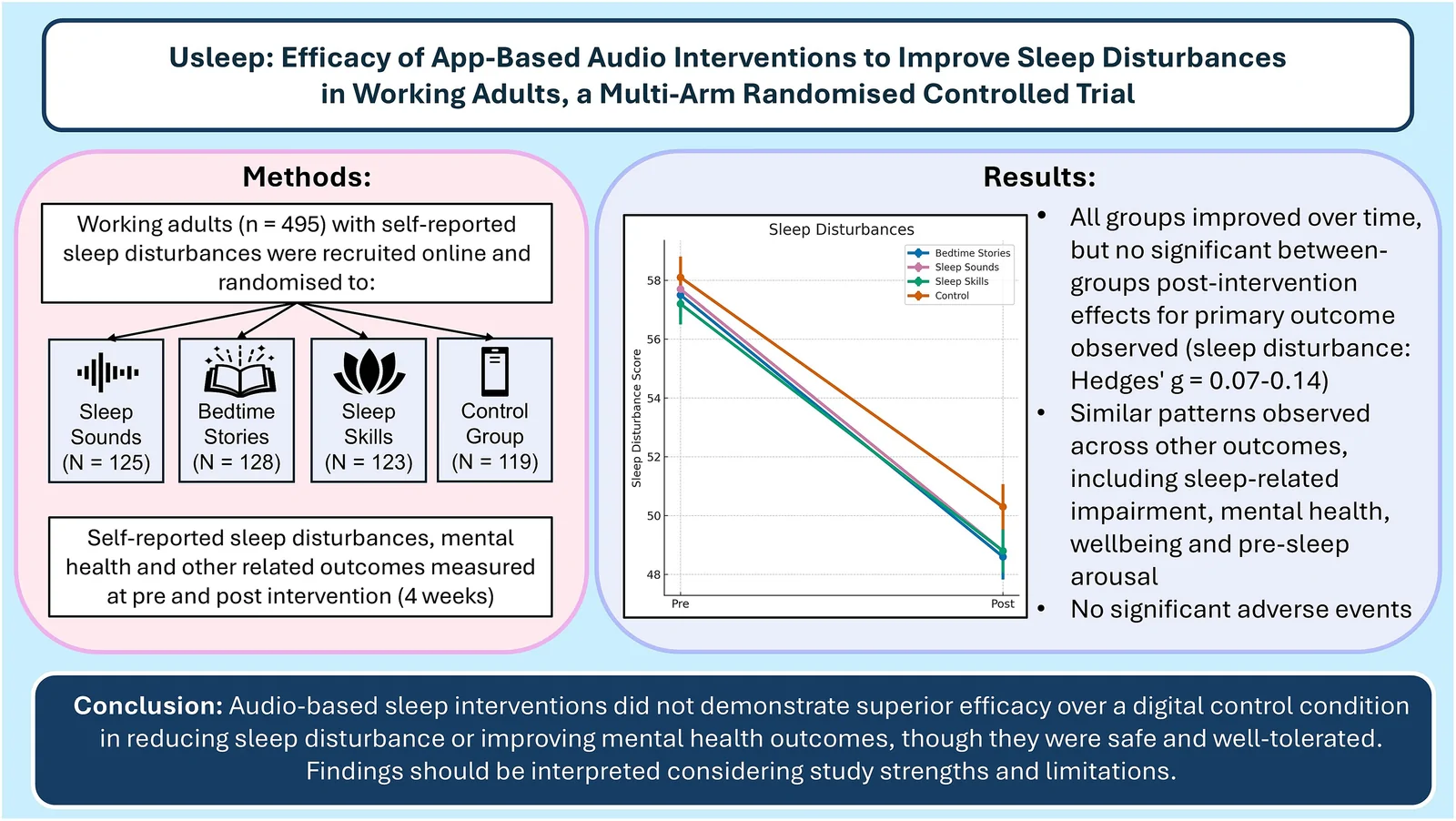

## Introduction

### Background and rationale

Sleep plays a crucial role in physical and mental health and social and occupational functioning [1–5]. Several dimensions contribute to sleep health, including adequate sleep duration, timing, regularity, satisfaction, alertness, and efficiency [6], each of which can be influenced by biological, behavioral, psychological, and environmental factors operating at the individual and societal level [7]. For example, insufficient sleep duration may result from limited opportunity for sleep, while sleep timing and regularity may be disrupted by shift work or irregular schedules [8, 9]. Conversely, many individuals struggle with their sleep despite adequate sleep opportunity, due to sleep disturbance [10]. This is the focus of the current study, and within this context, sleep disturbance is operationalized as a non-disorder-specific construct encompassing individuals’ perception of sleep quality, depth, and restorative value. This conceptualization includes clinical disorders such as insomnia, the most common sleep disorder, as well as subclinical presentations, such as difficulties initiating or maintaining sleep that do not meet formal diagnostic thresholds. Although ~16% of the population meets diagnostic criteria for insomnia [11], broader sleep disturbance has been estimated to affect 39.7% of working adults in the United Kingdom and 32.3% in the United States [12].

Effective treatments for sleep disturbance are available. Cognitive Behavioral Therapy for Insomnia (CBT-I) is the first-line treatment recommended by the National Institute for Health and Care Excellence [10] and is effective at treating insomnia [13, 14], including when delivered digitally via Sleepio (approved by the National Health Service) for both clinical [15] and subclinical insomnia [16]. However, professional help-seeking for sleep disturbance is low, particularly when the cause is perceived to be psychological [17], and many people turn to self-help instead [18], including direct-to-consumer apps [19]. Some of the most popular apps, downloaded by millions, offer standalone, audio-based sleep aids, including sleep music, colored noise, soundscapes, sleep stories, and sleep-adapted meditation, often combined to create sleep-inducing tracks [20]. Unlike CBT-I and other interventions, which focus on behavioral changes implemented throughout the day, these tools are designed specifically for the periliminal sleep period, the transitional phase before, and during the onset of sleep. For a synthesis of the evidence base and its limitations, see [20]. To date, only a single pilot randomized controlled trial (RCT) has assessed the efficacy of sleep sounds and narrated stories as a standalone intervention and delivered via a mental health app for self-reported sleep outcomes in working adults with sleep disturbance. Both interventions were found to be feasible and acceptable, with positive moderate to large effect sizes indicating improvements in sleep disturbance, sleep-related impairment, and other mental health and well-being outcomes compared to a waitlist control [21]. The lack of active control conditions is a common limitation in digital health research, partly because devising credible and appropriate controls is challenging [22, 23], especially in the context of sleep, which is influenced by numerous factors [24]. Nonetheless, comparing these audio tools with an active control is important to address these limitations and better understand the true magnitude of their effects. Furthermore, research investigating the underlying mechanisms of action of these tools is lacking. A conceptual framework previously proposed by our research group outlines several potential mechanisms, some shared and some unique to specific types of audio interventions, such as distraction, masking, or relaxation [20]. Therefore, there is a need to examine the efficacy of audio tools when compared with an active control group, and to better understand the potential mechanisms underlying these effects.

### Study objectives

The primary aim of the current study was to build on the results of a pilot study [21], and conduct a definitive, rigorous RCT to evaluate the efficacy of three categories of standalone, audio-based sleep exercises (“Sleep Tools”) delivered via a mental health phone app (MHapp). These categories were: (1) Bedtime Stories (BS; narrated stories), (2) Sleep Sounds (SS; ambient music, colored noise, and nature sounds), and (3) Sleep Skills (SK; guided mindfulness, relaxation, positive psychology, and breathwork practices). The first two categories, BS and SS, were included in the pilot study, while SK was novel and added to reflect the variety of content available online.

The primary outcome of interest was self-reported sleep disturbance. We hypothesized that there would be significant improvements in sleep disturbance from pre- to post-intervention for each Sleep Tool compared with the control group. We also expected to observe improvements in secondary outcomes, including sleep-related impairment, sleep duration, sleep onset latency, mental health, mental well-being, and workplace presenteeism. Given the paucity of evidence on the mechanisms involved, we further aimed to explore the effects of the Sleep Tools on pre-sleep arousal and cognitive emotion regulation, to investigate whether these variables are influenced by Sleep Tool use. Finally, the study aimed to assess the credibility and expectancy, feasibility and acceptability of each condition, adherence metrics, and self-reported adverse effects.

## Materials and methods

### Trial registration and ethical approval

The study was approved by the University of Sussex Science & Technology Research Ethics Committee (ER/JV268/2). It was pre-registered in the ISRCTN registry (registration reference: ISRCTN13426045) on October 11, 2024. The study adhered to the ethical principles of the Declaration of Helsinki, and all participants provided informed consent.

### Design and settings

The study followed a multi-arm, parallel-group RCT design with assessments conducted at t0 (pre-intervention) and t1 (post-intervention, 4 weeks later). Participants were randomly assigned in a 1:1:1:1 ratio to one of three Sleep Tools interventions (BS, SS, or SK) or to a control group Daily Boost (DB), all delivered via a MHapp (Unmind) for a duration of 4 weeks. The study was prospectively registered on the International Standard Randomized Controlled Trial Number (ISRCTN) (registration number: 13426045). No patient or public involvement was included in the design, conduct, or reporting of this trial. No changes were made to the planned outcomes or analyses, although recruitment ended before the target sample size was reached for budgetary reasons.

### Participants

Screening opened on October 22, 2024, and participant enrolment for the main trial began on October 28, 2024 and terminated on November 27, 2024. Study participants were recruited via Prolific (https://www.prolific.co) [25]. Inclusion criteria were: (a) working age (18–67 years), (b) current residence in the United Kingdom or United States, (c) self-identifying as employed full- or part-time, (d) self-reported poor sleep (scoring ≥7 on the Jenkins Sleep Scale [26]), (e) self-reporting an interest in improving sleep, (f) self-reported fluency in English, (g) access to the Internet at home via a smartphone, laptop, desktop, or tablet, (h) ownership of a smartphone (and/or tablet) and willingness to download and use a MHapp, (i) willingness to be randomized, and (j) having an active Prolific account. Exclusion criteria included: (a) receiving treatment for a sleep disorder from a healthcare professional and (b) previous or current use of the MHapp.

### Sample size

The study was powered to detect effects on the primary contrast of interest: the individual comparison of each intervention arm to the control condition on Sleep Disturbance, measured using the PROMIS Sleep Disturbance Short Form (SD-SF) from t0 to t1.

Effect sizes on the primary outcome were estimated to be small based on previous evidence of the effectiveness of Sleep Tools against wait list controls [21, 27] and the influence of active control conditions [28]. Taken together, power analysis indicated that detecting an estimated small effect (Hedges’ *g* = 0.25) on change in SD-SF, with 80% power at an alpha level of 0.05, required a minimum sample of 520 participants.

Considering that 17% of participants in the pilot study either did not start the intervention after randomization or were lost to follow-up [21], and taking financial constraints into account, we aimed to recruit 600 participants. Based on pilot study eligibility rates, study uptake, and financial considerations, it was estimated that screening 1000 participants would be necessary to achieve the desired sample size. No interim analyses or formal stopping guidelines were pre-specified.

### Procedure and randomization

An advertisement for the study was posted on Prolific to eligible participants, who were pre-screened through the platform. To increase the generalizability of the results to a broad population of people experiencing sleep disturbance and ensure a balanced sample, four advertisements were created, targeting different working age groups (18–25, 26–67) based on UK and US Census data, as well as geographical locations (United Kingdom and United States). Each group had an additional requirement for a 50/50 gender split, as previous research is based on predominantly female samples [21, 27, 29, 30]. Participants were pre-screened via Prolific based on employment status, age, geographical location, and their eligibility was further assessed via a short screening self-report questionnaire based on inclusion and exclusion criteria. Eligible participants were invited to take part in the study through Prolific on a first-come, first-served basis, and were asked to complete the baseline questionnaire. Prolific was integrated with the online survey platform Qualtrics (https://www.qualtrics.com), where all questionnaires were hosted.

The baseline assessment consisted of four main parts. Participants first viewed an information sheet and a brief video detailing the study design. Comprehension of key instructions was assessed via a four-question quiz; incorrect responses prompted a replay of the video and a retest, followed by the display of correct answers. Subsequently, participants provided informed consent (Part 1). Consenting participants reported demographic information and completed seven psychometric scales, presented in random order (Part 2). Participants were then randomly assigned to one of the four interventions (1:1:1:1 allocation ratio) using Qualtrics block randomization. Although researchers were not involved in the randomization process, they were not blinded to group allocation during the study or data analysis. Participants were informed of their group allocation and viewed a video with instructions on engaging with their assigned feature and accessing content on the app (Part 3), although those in the DB group were not explicitly informed that they had been assigned to the control condition. No concomitant care or co-interventions were provided as part of the trial beyond the allocated app-based condition. Finally, participants completed a brief questionnaire to assess their credibility and expectations regarding their allocated intervention (Part 4).

Written instructions were also sent via Prolific’s anonymous messaging system, along with weekly reminders about their participation in the study. Engagement with the app was encouraged but not mandatory. Post-intervention surveys were administered after the 4-week period.

To facilitate recruitment and retention, participants received £1 for the screening and £7 for completing each of the study assessments (t0 and t1), which were each estimated to take ~30 min to complete. This resulted in a maximum possible total payment of £15 (or the equivalent in $USD).

### Sleep interventions

The sleep interventions reflected categories of popular standalone, audio-based tools used as sleep aids, like those offered by a range of widely used sleep apps or found on streaming platforms and examined in previous research [30–35]. A detailed description of intervention components is provided in the Template for Intervention Description and Replication (TiDiER) checklist in the Supplementary Material.

Participants were asked to engage with one Sleep Tool per night in preparation for sleep, while falling asleep, throughout the night, or when returning to sleep after waking during the night, consistent with user accounts of how such tools are typically used in real-world settings [30]. They could listen to the same track each night or choose a different one, depending on their preferences or desire for variety. It was made clear that missing a night or two was acceptable, and no minimum duration of listening was prescribed, to resemble every day, self-directed use of such interventions as closely as possible. The tools were accessed via a media player that allowed participants to pause, loop, or close the content.

### Sleep sounds

This category comprised 60 audio tracks without voice narration (length: 5–60 min), including music, colored noise, nature sounds, and tracks containing a combination of these elements. Music tracks featured genres including classical, ambient, and jazz, but all shared typical sleep music characteristics: slow tempos (60–85 bpm), minor tonalities, smooth melodies, and an absence of dramatic changes in volume or rhythm [31]. The library also included white and pink noise tracks.

### Bedtime stories

This category comprised 18 narrated stories in English (length: 15–43 min) focused on descriptive, low-arousal narratives, sometimes accompanied by background soundscapes or brief relaxation prompts. All stories were delivered in softly spoken, calming voices with warm tones.

### Sleep skills

This category comprised 8 voice-led audio exercises (length: 10–16 min). Content included sleep hygiene and bedtime-specific behavioral recommendations, mindfulness practices, progressive muscle relaxation, breathing exercises, and two positive psychology practices, one aimed at eliciting gratitude and the other at promoting self-compassion. The tracks were voice-only, evenly split between two narrators, one male and one female, both using neutral, slightly soft tones that remained consistent throughout.

### Control condition

The digital control condition involved engagement with a separate feature of the same MHapp, called the DB. The DB consists of standalone tools that change daily, offering a variety of content, including brief psychoeducation on various aspects of health and well-being, reflection or relaxation prompts, and poetry. The DB is not designed to specifically support sleep health. Participants could choose to engage with the DB by either listening to the audio version or reading the script, both of which were accessible via the same media player used in the intervention. The digital control condition was matched to the experimental conditions on access to the same mental health app and related user experience, use frequency, number of sessions, and trial duration.

### Outcomes measures

#### Primary outcome measures

##### PROMIS Sleep Disturbance Short Form 8a

The SD-SF is a widely used 8-item measure assessing self-reported sleep disturbance, perceived sleep quality, and satisfaction over a 7-day recall period [36]. Participants respond using a 5-point Likert scale, with total scores ranging from 8 to 40, which are then converted into standardized T-scores (mean = 50, *SD* = 10). The scale is adapted from the original 27-item version [37] and demonstrates strong psychometric properties, including high reliability (≥.90 for θ scores between −1.5 and 2.5) [36].

#### Secondary outcome measures

Secondary outcome measures were pre-registered and assessed the effectiveness of each intervention arm compared to the control condition across various sleep, mental health, and well-being outcomes using psychometric scales. Additionally, secondary measures included feedback and adherence outcomes (objectively measured) to evaluate feasibility, acceptability, satisfaction, relevance, and engagement across all study arms.

##### PROMIS Sleep-Related Impairment Short Form 8a

The Sleep-Related Impairment Short Form 8a (SRI-SF) assesses the impact of poor sleep on daily functioning, including difficulties with alertness, tiredness, sleepiness, and productivity over a 7-day recall period [36]. It was developed from the original 16-item version [37] alongside the SD-SF and shares many of its characteristics, including its 8-item format and universal applicability across various conditions. The response options, recall period, scoring conversions, and interpretation are as described for the SD-SF. The SRI-SF has demonstrated high reliability (≥.90) [36].

##### Sleep latency and duration

Two items from the Medical Outcomes Study Sleep Scale (MOS-SS [38]) were included in the study, to self-report (1) average time taken to fall asleep, and (2) average number of hours of sleep per night. Both items have a 4-week recall period.

##### Patient Health Questionnaire-4

The Patient Health Questio-nnaire-4 (PHQ-4) is a widely used brief self-report measure designed to assess core symptoms of anxiety (items 1 and 2) and depression (items 3 and 4) [39]. Respondents rate the frequency of symptoms over the past 2 weeks on a 4-point scale (0 = “Not at all” to 3 = “Nearly every day”). The total score ranges from 0 to 12, with higher scores indicating greater symptom severity. Scores are classified as follows: normal (0–2), mild (3–5), moderate (6–8), and severe (9–12). Its reliability has been validated as a screening tool for depression and anxiety symptoms in both clinical and nonclinical populations [40].

##### Short Warwick-Edinburgh Mental Well-Being Scale

The Short Warwick-Edinburgh Mental Well-Being Scale (SWEMWBS) is a scale designed to assess mental well-being, with a focus on functioning, using a 2-week recall period [41]. This scale is a shorter version (7 items) of the original 14-item Warwick-Edinburgh Mental Well-being Scale ([42]). Respondents rate the frequency of how often they experience the feelings described in each statement on a 5-point scale, ranging from 1 (“None of the time”) to 5 (“All of the time”). Raw scores are converted into metric scores, ranging from 7 to 35, with higher scores indicating greater mental well-being. The SWEMWBS has demonstrated high internal consistency (Cronbach’s alpha = .84 in the UK general population) [43].

##### Work Productivity and Activity Impairment

The Work Productivity and Activity Impairment (WPAI) scale is a widely used self-report tool that measures the impact of health problems on work productivity and daily activities, with a 1-week recall period [44]. The version used in the current study is adapted to focus on a specific health problem (WPAI:SHP), with respondents answering questions related to impairment due to “poor sleep.” It consists of six items that assess absenteeism, presenteeism, work productivity loss, and activity impairment. The total scores are expressed as percentages, with higher percentages indicating greater impairment. Due to the structure of the scale, internal consistency cannot be calculated; however, the scale demonstrates good test–retest reliability [44].

##### Pre-Sleep Arousal Scale

The Pre-Sleep Arousal Scale (PSAS) is a 16-item scale aimed at evaluating states of arousal when attempting to fall asleep [45]. The scale features two eight-item subscales, one encompassing cognitive aspects (e.g. Worry about falling asleep) and another focusing on somatic elements (e.g. Shortness of Breath). Respondents are asked to rate each item on a 5-point Likert scale from 1 (“Not at all”) to 5 (“Extremely”). Total scores range from 8 to 40, with higher scores indicating elevated pre-sleep cognitive and/or somatic arousal. The validity and reliability of the cognitive and somatic subscales of the PSAS are reported as α = 0.76 and α = 0.81, respectively [45].

##### Cognitive Emotion Regulation Questionnaire-18 items version

The Cognitive Emotion Regulation Questionnaire (CERQ-short) is a shorter, 18-item version of the CERQ (36 items) designed to assess cognitive emotion regulation strategies in response to negative events [46]. It includes nine subscales, each represented by two items rated on a 5-point Likert scale 1 (“Almost never”) to 5 (“Almost always”). Subscales for maladaptive strategies (self-blame, other-blame, rumination, catastrophising) and adaptive strategies (acceptance, positive refocusing, planning, positive reappraisal, putting into perspective) are calculated by averaging the relevant items. Higher scores indicate greater use of the respective strategy type. CERQ-short scale has shown to have good psychometric properties, while maintaining the same subsections as the original version [46].

#### Other measures

##### Jenkins Sleep Quality Scale

The Jenkins Sleep Quality Scale (JSS) is a brief 4-item tool that assesses the frequency and intensity of common sleep difficulties [26]. Participants are asked to report how often they experience specific sleep disturbance over the past month, with responses given on a 5-point Likert scale ranging from 0 = Not at all to 5 = 22–31 days. Total scores range from 0 to 20, with higher scores indicating greater sleep disturbance. While a higher threshold (≥11) has been used in research to indicate more severe or frequent sleep disturbance [47], a cutoff score of ≥7 was used in the current study to include milder presentations. Research suggests a mean score of 3.83 in the general population (*SD* = 4.48), and that a cut-off point of 7 captures the top quintile (20%) of sleep disturbance [48] and was used in the preceding pilot study [21].

##### Credibility/Expectancy Questionnaire-6

The Credibility/Expectancy Questionnaire-6 (CEQ-6) is a brief, 6-item measure that evaluates the credibility and expectancy of treatment [49]. The original scale was devised to assess credibility and expectancy of therapy. The version employed in the current study is adapted to match the intervention used and the primary outcome (reduction in sleep disturbance). Examples of items include “How confident would you be in recommending this app feature to a friend who experiences similar problems?” and “By the end of the study period, how much improvement in your sleep disturbances do you think will occur?” Respondents are asked to rate four items on a 9-point scale ranging from 1 (“Not at all”) to 9 (“Very much”), and two items from 0% to 100%. Total scores are standardized via conversion to a *z*-score, and higher scores are associated with higher credibility and/or expectancy. The CEQ has strong psychometric properties, including good internal consistency (Cronbach’s alpha = .85) [49].

##### Demographic Questionnaire, Feedback Questionnaire, and Objective Engagement

Participants were presented with a demographic questionnaire (t0), a feedback questionnaire at t1 aimed at capturing satisfaction and other feasibility outcomes, as well as self-reported engagement and harm. Harm was defined based on participants’ agreement or disagreement (Likert scale) with the statement: “I experienced lasting bad effects from use of the Unmind Sleep Tools,” with an additional open-text field allowing participants to describe any lasting adverse effects. Engagement data were objectively captured through the platform’s built-in usage tracking. See Appendix Measures S1 and S2 for details.

### Statistical analysis

All statistical analyses were performed using R (R Core Team, 2025). The demographic and other baseline characteristics were analyzed and reported via descriptive statistics (frequencies, means, and standard deviations).

#### Statistical analyses for primary and secondary outcomes

All primary and secondary outcomes were analyzed using both an intention-to-treat (ITT) and per protocol (PP) approach, except for the feedback survey answers and the credibility and expectancy questionnaire that were analyzed for ITT only. The ITT analysis included all eligible participants who provided a valid response at t0, regardless of their intervention engagement or dropout status. The PP approach included only participants who engaged with their allocated feature at least 12 times in total across the study period.

In both analyses, the primary contrast of interest compared each experimental arm (BS, SS, SK) to the control condition (DB). Additionally, global tests comparing the pooled intervention arms combined (Sleep Tools) to the control condition were also conducted. *p* values <.05 were considered significant.

#### Statistical analysis for primary outcomes

Linear mixed-effects models (LMEs) with maximum likelihood estimation, using the lmer function from the lme4 package in R [50] were used to assess changes in SD-SF from t0 to t1. LMEs account for missing data under the assumption that data are missing at random, allowing all available observations to contribute to the analysis. The model included a between-group factor (with BS, SS, SK each compared to the control group set as the reference), and a within-participant factor (time point: with levels t0 (Pre) and t1 (Post)). A random intercept for each participant (based on their unique IDs) was included, controlling for individual variability at baseline. To assess the overall intervention effect, the same model was run with all intervention arms combined (Sleep Tools) and tested against the control (DB). Model residuals were examined using Q–Q and residual–fitted plots to assess model assumptions and fit; only minor deviations at the upper tail were observed. Estimated marginal means (EMMs) with standard errors (*SE*s) were reported for each group and time point, as well as for the combined intervention arms. Within-group changes (t0 − t1) were presented along with the corresponding *p*-values. For between-group comparisons, *p*-values, Hedges’ *g*, and 95% confidence intervals were reported. Hedges’ *g* was calculated using EMMs and pooled standard deviations, as in the pilot study [21].

#### Statistical analysis for secondary outcomes

##### Pre-post changes of outcome measures

The same LME models were used to assess pre-post changes in all continuous, questionnaire-based secondary outcomes, following the same reporting approach as the primary outcome.

For sleep onset latency, which had five response categories, *n* and % were calculated for each category and reported in a table. For the logistic regression analysis, these five categories were collapsed into two levels: participants who reported falling asleep in less than 30 min and those who reported taking more than 30 min. A binomial logistic regression was then performed to examine changes between t0 and t1, with Group, Time, and their interaction included as predictors to assess between-group differences over time.

##### Single-time point outcome measures

Categorical outcome variables, such as responses to the feedback questionnaire, were reported as the number of participants in each category along with the corresponding percentages. Fisher’s exact test (two-tailed) was used to determine significant differences between the combined intervention groups (Sleep Tools) and the active control condition (DB), with the associated *p*-value reported. For comparisons between BS and SS, refer to the pilot study [21].

Differences in credibility and expectancy (CEQ-6) between groups were investigated using one-way analysis of variances (ANOVAs). In case of significant results, pairwise comparisons with post-hoc Tukey’s Honest Significant Difference (HSD) were performed to identify the specific group differences.

##### Objective engagement outcome measures

Engagement data were objectively captured through the platform’s built-in usage tracking and linked to participant responses using a unique ID. From the raw data, several variables were extracted. *Individual Sessions Opened* (ISO) referred to any intervention session accessed, regardless of its completion. *Individual Sessions Completed* (ISC) included any session of the allocated intervention that participants fully completed. For Sleep Tools, completion was defined as playing a track for 5 min or longer, acknowledging the flexible nature of these tools (e.g. if a participant felt sleepy after a short period of use).

Given the differences in length between the intervention arms and the DB, additional metrics were applied specifically to the Sleep Tools. *Sessions Based on Duration* measured the number of sessions played, accounting for instances where participants played sessions on a loop throughout the night. Lastly, *Total Playtime* (measured in hours) represented the total time participants spent engaging with the Sleep Tools.

Independent sample *t*-tests were conducted to assess differences between the combined intervention arms (Sleep Tools) and the DB for ISO and ISC. One-way ANOVAs were performed to compare between groups, followed by post-hoc analysis using Tukey HSD when significant. Similarly, one-way ANOVAs of Sleep Tools were conducted for Sessions Based on Duration and Total Playtime, with pairwise comparisons performed where necessary. Finally, from the raw data, participants who engaged with content outside of their allocated condition were identified to assess compliance.

### Exploratory analyses

Planned exploratory analyses aimed to investigate potential mechanisms of action by running LMEs (as described above for primary outcomes) on pre-sleep arousal (PSA) and cognitive emotion regulation (CERQ).

Further unplanned exploratory analyses were conducted to better understand study results. A sensitivity analysis using multiple imputation (30 imputed datasets, predictive mean matching method) was conducted to handle missing follow-up data from dropouts, with results compared to ITT analysis. To explore whether baseline sleep disturbance moderated the effect of the intervention on changes in sleep disturbance, and whether this effect differed between groups, a moderated multiple regression analysis was performed. Finally, linear regression models were used to investigate whether the number of ISC predicted changes in sleep disturbance for the control condition and the sleep intervention groups (analyzed separately).

## Results

### Participants

**Figure 1:**
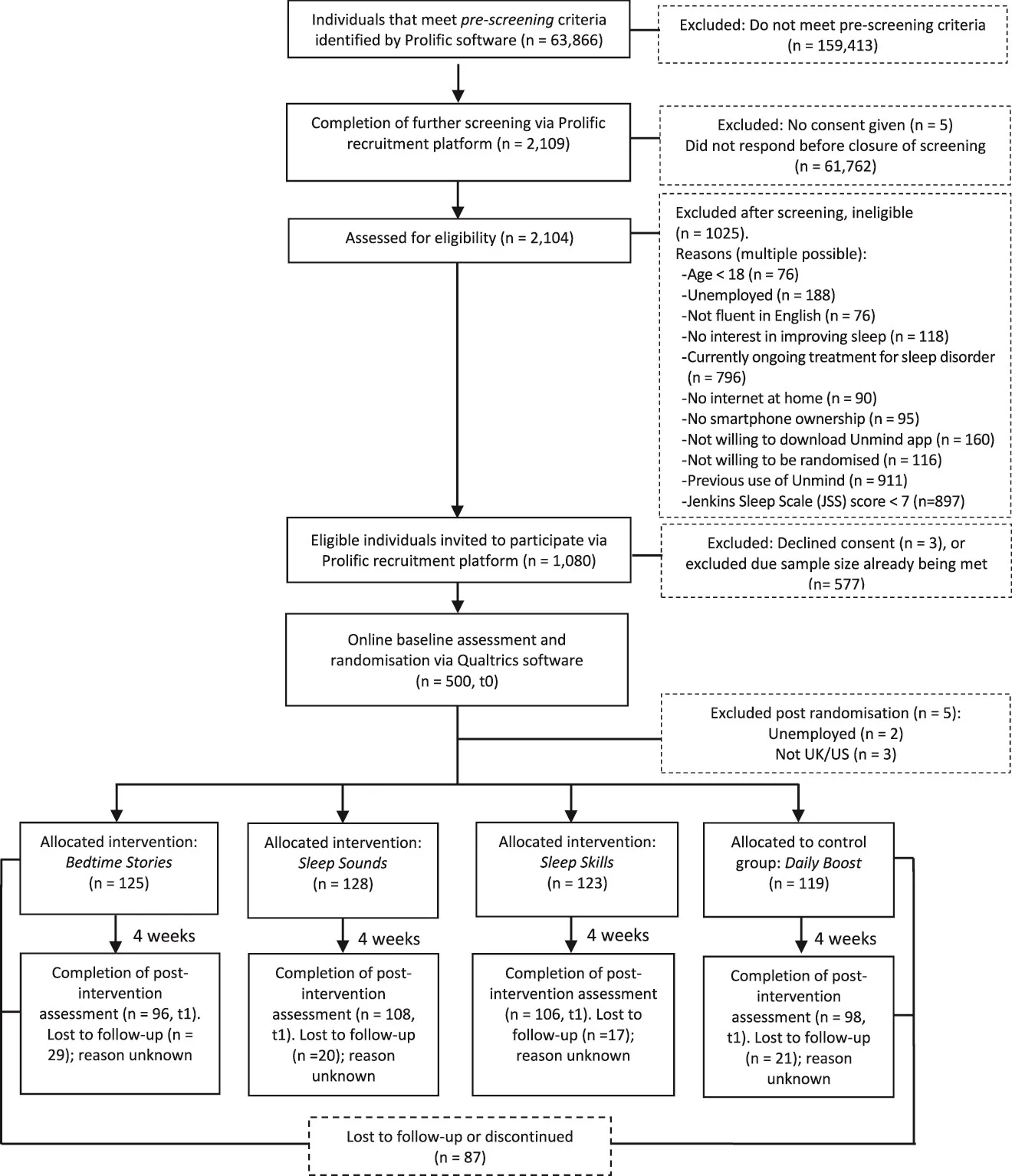
CONSORT chart of participant flow through the study.

Figure 1 shows the participant flow throughout the trial. A total of 2109 individuals were screened, of whom 1080 (51.2%) met the eligibility criteria. Eligibility rates were lower than anticipated based on the pilot study [21], resulting in higher-than-expected screening costs. Consequently, recruitment was stopped at 500 (83.3% of the target sample) participants for budgetary reasons. Of these, 495 were enrolled in the trial. A total of 87 participants were lost to follow-up, resulting in a retention rate of 82.4%. The sample included 61.2% participants from the United Kingdom and 38.8% from the United States. At baseline, the mean PROMIS SD-SF score was 57.60 (*SD* = 7.07). Participants slept on average 6 h and 14 min (*SD* = 1 h and 17 min). A total of 293 participants (59.2%) reported a sleep onset latency of more than 30 min, with 16.0% reporting more than an hour. Moreover, 69.5% of participants scored above the PHQ-4 cutoff (≥3), indicating at least mild symptoms of anxiety and/or depression [51], and the mean mental well-being score was 20.37 (*SD* = 3.86). 9.9% of the sample reported a past sleep diagnosis, with a higher proportion from the United States (16.7%) compared to the United Kingdom (5.6%). However, an independent *t-*test of all questionnaire-based outcomes revealed no significant differences in sleep or mental health between participants from the United States and United Kingdom, except for CERQ total adaptive cognitive emotion regulation skills, with US participants demonstrating significantly higher scores than their UK counterparts (see supplementary material, Table S1). Further demographic details are presented in Table 1.

**Table 1:** Demographic variables and sample characteristics

Variable	Overall (*n* = 495)	Experimental arms			Control
		Bedtime Stories (*n* = 125)	Sleep Sounds (*n* = 128)	Sleep Skills (*n* = 123)	Daily Boost (*n* = 119)
**Age (years)**					
Mean (*SD*)	32.7 (12.00)	32.83 (11.93)	32.91 (12.24)	32.46 (11.47)	32.97 (12.48)
Range	18-67	18-66	18-65	18-62	18-67
**Sex assigned at birth, *n* (%)**					
Female	276 (55.76)	69 (55.20)	79 (61.72)	69 (56.10)	59 (49.58)
Male	219 (44.24)	56 (44.80)	49 (38.28)	54 (43.90)	60 (50.42)
**Gender identity, *n* (%)**					
Female	269 (54.34)	67 (53.60)	78 (60.94)	66 (53.66)	58 (48.74)
Male	214 (43.23)	55 (44.00)	48 (37.50)	54 (43.90)	57 (47.90)
Non-binary	8 (1.62)	2 (1.60)	2 (1.56)	1 (0.81)	3 (2.52)
Transgender	4 (0.81)	1 (0.80)	0 (0.00)	2 (1.63)	1 (0.84)
**Ethnicity, *n* (%)**					
White	351 (70.91)	89 (71.20)	92 (71.88)	85 (69.11)	85 (71.43)
Black	53 (10.71)	10 (8.00)	15 (11.72)	14 (11.38)	14 (11.76)
Asian	49 (9.90)	13 (10.40)	15 (11.72)	14 (11.38)	7 (5.88)
Hispanic or Latino	15 (3.03)	3 (2.40)	3 (2.34)	4 (3.25)	5 (4.20)
Mixed	24 (4.85)	10 (8.00)	3 (2.34)	6 (4.88)	5 (4.20)
Other	3 (0.61)	0 (0.00)	0 (0.00)	0 (0.00)	3 (5.52)
**Education, *n* (%)**					
No qualification	1 (0.20)	0 (0.00)	0 (0.00)	0 (0.00)	1 (0.84)
High School	197 (39.80)	50 (40.00)	49 (38.28)	42 (34.15)	56 (47.06)
University degree	217 (43.84)	49 (39.20)	60 (46.88)	61 (49.59)	47 (39.50)
Postgraduate degree	80 (16.16)	26 (20.80)	19 (14.82)	20 (16.26)	15 (12.61)
**Employment status, *n* (%)**					
Full-time employee	336 (67.88)	83 (66.40)	88 (68.75)	87 (70.73)	78 (65.55)
Part-time employee	116 (23.43)	28 (22.40)	34 (26.56)	26 (21.24)	28 (23.53)
Self-employed/contractor	43 (8.69)	14 (11.20)	6 (4.69)	10 (8.13)	13 (10.92)
**Employment industry, *n* (%)**					
Agriculture/forestry/mining	2 (0.40)	1 (0.80)	0 (0.00)	1 (0.81)	0 (0.00)
Industrials	27 (5.45)	5 (4.00)	6 (4.69)	6 (4.88)	10 (8.40)
Energy, utilities	4 (0.81)	1 (0.80)	0 (0.00)	3 (2.44)	0 (0.00)
Transport, logistics	15 (3.03)	5 (4.00)	1 (0.78)	3 (2.44)	6 (5.04)
Media, creative industries	18 (3.64)	6 (4.80)	3 (2.34)	4 (3.25)	5 (4.20)
Data, telecom	19 (3.84)	5 (4.00)	5 (3.91)	4 (3.25)	5 (4.20)
Healthcare	52 (10.51)	10 (8.00)	19 (14.84)	9 (7.32)	14 (11.76)
Education	65 (13.13)	18 (14.40)	14 (10.94)	17 (13.82)	16 (13.45)
Life sciences	7 (1.41)	0 (0.00)	3 (2.34)	4 (3.25)	0 (0.00)
Retail, E-commerce	54 (10.91)	16 (12.80)	15 (11.72)	9 (7.32)	14 (11.76)
Hospitality, food, leisure, travel	35 (7.07)	9 (7.20)	10 (7.81)	10 (8.13)	6 (5.04)
Public service, social service	46 (9.29)	11 (8.80)	18 (14.06)	10 (8.13)	7 (5.88)
Finance, insurance, real estate	38 (7.68)	8 (6.40)	5 (3.91)	14 (11.38)	11 (9.24)
Professional services	38 (7.68)	11 (8.80)	11 (8.59)	7 (5.69)	9 (7.56)
Other	75 (15.15)	19 (15.20)	18 (14.06)	22 (17.89)	16 (13.45)
**Work arrangements, *n* (%)**					
Working from home	110 (22.20)	25 (20.00)	22 (17.19)	33 (26.83)	30 (25.21)
Working in the office	101 (20.40)	29 (23.20)	24 (18.75)	25 (20.33)	23 (19.33)
Hybrid	183 (36.97)	46 (36.80)	49 (38.28)	47 (38.12)	41 (34.45)
On-site working	101 (20.40)	25 (20.00)	33 (25.78)	18 (14.63)	25 (21.01)
**Long-term leave, *n* (%)**					
Sick leave	4 (0.81)	0 (0.00)	3 (2.34)	0 (0.00)	1 (0.84)
Other leave	5 (1.01)	1 (0.80)	2 (1.56)	1 (0.81)	1 (0.84)
No	486 (98.18)	124 (99.20)	123 (96.09)	122 (99.19)	117 (98.32)
**Perform shift work, *n* (%)**					
Yes	57 (11.52)	18 (14.40)	8 (6.25)	18 (14.63)	13 (10.92)
No	438 (88.48)	107 (85.60)	120 (93.75)	105 (85.37)	106 (89.08)
**Children in care, *n* (%)**					
Yes	117 (23.64)	33 (26.40)	23 (17.97)	32 (26.02)	29 (24.37)
No	378 (76.36)	92 (73.60)	105 (82.03)	91 (73.98)	90 (75.63)
**Bed partner arrangements, *n* (%)**					
No bed partner	195 (39.39)	42 (33.60)	55 (42.97)	53 (43.09)	45 (37.82)
Separate beds	64 (12.93)	15 (12.00)	16 (12.50)	14 (11.38)	19 (15.97)
Same bed	236 (47.68)	68 (54.40)	57 (44.53)	56 (45.53)	55 (46.22)
**Attended therapy (last 6 months), *n* (%)**					
Yes	52 (10.51)	13 (10.40)	17 (13.28)	8 (6.50)	14 (11.76)
No	443 (89.49)	112 (89.60)	111 (86.72)	115 (93.50)	105 (88.24)
**Mental health app use (last 6 months), *n* (%)**					
Yes	54 (10.91)	16 (12.80)	14 (10.94)	13 (10.57)	11 (9.24)
No	441 (89.09)	109 (87.20)	114 (89.06)	110 (89.43)	108 (90.76)
**Use of sleep remedies, *n* (%)**					
Yes	53 (10.71)	14 (11.20)	22 (17.19)	6 (4.88)	11 (9.24)
No	442 (89.29)	111 (88.80)	106 (82.81)	117 (95.12)	108 (90.76)
**Past sleep diagnosis, *n* (%)**					
Yes	49 (9.9)	17 (13.60)	15 (11.72)	8 (6.50)	9 (7.56)
No	446 (90.1)	108 (86.40)	113 (88.28)	115 (93.50)	110 (92.44)
**Alcohol intake, *n* (%)**					
Never	142 (28.69)	34 (27.20)	39 (30.47)	34 (27.64)	35 (29.41)
Monthly or less	145 (29.29)	36 (28.80)	39 (30.47)	37 (30.08)	33 (22.73)
Several per month	130 (26.26)	31 (24.80)	32 (25.00)	34 (27.64)	33 (22.73)
Several per week	64 (12.93)	18 (14.40)	17 (13.28)	16 (13.01)	13 (10.92)
Almost daily	14 (2.83)	6 (4.80)	1 (0.78)	2 (1.63)	5 (4.20)
**Other factors impacting sleep, *n* (%)**					
Yes	170 (34.34)	51 (40.80)	41 (32.03)	33 (26.83)	45 (37.82)
No	325 (65.66)	74 (59.20)	87 (67.97)	90 (73.17)	74 (62.18)

### Primary outcome

Table 2 presents the EMMs for each group, as well as for all intervention arms combined (Sleep Tools) at t0 and t1, the difference (t0 − t1), the *p*-value for within-group contrasts, and between-group values and Hedges’ *g* with 95% confidence intervals based on between-group contrasts, with DB set as the reference category. Raw means and standard errors for all outcome measures can be found in the supplementary material, Table S2.

**Table 2:** Estimated marginal means (ITT sample) for linear mixed-effects models with standardized effect sizes

Outcome	Estimates (mean, *SE*)			*p* value (within-group)	*p* value (between-group)	Hedges’ *g* [95% CI] (between-group)
	t0	t1	t0 − t1			
**SD-SF**						
Daily Boost	58.1 (0.71)	50.3 (0.77)	7.78 (0.80)	<.001		
Bedtime Stories	57.5 (0.69)	48.6 (0.77)	8.85 (0.80)	<.001	.346	0.12 [−0.13, 0.37]
Sleep Sounds	57.5 (0.69)	48.6 (0.73)	8.89 (0.76)	<.001	.281	0.14 [−0.11, 0.39]
Sleep Skills	57.4 (0.70)	49.0 (0.74)	8.38 (0.77)	<.001	.594	0.07 [−0.18, 0.32]
Sleep Tools	57.5 (0.40)	48.7 (0.43)	8.73 (0.45)	<.001	.300	0.11 [−0.07, 0.29]
**SRI-SF**						
Daily Boost	60.9 (0.80)	53.5 (0.86)	7.37 (0.84)	<.001		
Bedtime Stories	61.0 (0.78)	51.5 (0.86)	9.53 (0.85)	<.001	.072	0.23 [−0.02, 0.48]
Sleep Sounds	60.9 (0.77)	52.5 (0.82)	8.31 (0.81)	<.001	.418	0.10 [−0.15, 0.35]
Sleep Skills	60.9 (0.79)	51.4 (0.83)	9.46 (0.82)	<.001	.075	0.23 [−0.02, 0.48]
Sleep Tools	60.9 (0.45)	51.8 (0.48)	9.08 (0.48)	<.001	.077	0.19 [−0.01, 0.36]
**Sleep duration** ^*^						
Daily Boost	6.08 (0.11)	6.64 (0.12)	0.55 (0.11)	<.001		
Bedtime Stories	6.12 (0.11)	7.00 (0.12)	0.88 (0.11)	<.001	.042	−0.26 [−0.51, −0.01]
Sleep Sounds	6.27 (0.11)	6.99 (0.11)	0.72 (0.11)	<.001	.280	−0.14 [−0.39, 0.11]
Sleep Skills	6.46 (0.11)	6.88 (0.12)	0.42 (0.11)	<.001	.393	0.11 [−0.14, 0.36]
Sleep Tools	6.28 (0.06)	6.95 (0.07)	0.67 (0.06)	<.001	.374	−0.09 [−0.27, 0.08]
**PHQ-4**						
Daily Boost	4.45 (0.29)	3.18 (0.31)	1.28 (0.26)	<.001		
Bedtime Stories	4.86 (0.29)	3.05 (0.31)	1.81 (0.26)	<.001	.145	0.19 [−0.07, 0.44]
Sleep Sounds	4.49 (0.28)	3.26 (0.30)	1.68 (0.25)	<.001	.266	0.14 [−0.11, 0.39]
Sleep Skills	4.39 (0.29)	3.31 (0.30)	1.08 (0.25)	<.001	.580	−0.07, [−0.32, 0.18]
Sleep Tools	4.73 (0.17)	3.22 (0.18)	1.52 (0.15)	<.001	.423	0.08 [−0.09, 0.26]
**SWEMWBS** ^*^						
Daily Boost	20.3 (0.38)	21.9 (0.41)	1.59 (0.38)	<.001		
Bedtime Stories	20.9 (0.37)	23.2 (0.41)	2.36 (0.39)	<.001	.158	−0.18 [−043, 0.07]
Sleep Sounds	19.9 (0.36)	22.0 (0.39)	2.11 (0.37)	<.001	.328	−0.12 [−0.38, 0.13]
Sleep Skills	20.5 (0.37)	21.8 (0.39)	1.30 (0.37)	<.001	.586	0.07 [−00.18, 0.32]
Sleep Tools	20.4 (0.21)	22.3 (0.23)	1.91 (0.22)	<.001	.477	−0.07 [0.10, 0.25]
**WPAI**						
**Absenteeism**						
Daily Boost	5.93 (0.80)	3.35 (0.88)	2.58 (0.99)	.009		
Bedtime Stories	5.27 (0.78)	1.96 (0.89)	3.31 (0.99)	.001	.603	0.07 [−0.19, 0.32]
Sleep Sounds	4.19 (0.78)	2.19 (0.85)	2.01 (0.96)	.037	.676	−0.05 [−0.30, 0.20]
Sleep Skills	4.11 (0.79)	2.50 (0.85)	1.61 (0.96)	.094	.481	−0.09 [−0.34, 0.16]
Sleep Tools	4.53 (0.45)	2.23 (0.50)	2.30 (0.56)	<.001	.802	−0.03 [−0.20, 0.15]
**Presenteeism**						
Daily Boost	44.6 (2.00)	29.7 (2.20)	14.9 (2.29)	<.001		
Bedtime Stories	46.5 (1.94)	27.0 (2.19)	19.5 (2.27)	<.001	.154	0.18 [−0.07, 0.44]
Sleep Sounds	44.4 (1.95)	26.4 (2.10)	18.0 (2.20)	<.001	.331	0.12 [−0.13, 0.37]
Sleep Skills	43.6 (1.96)	25.4 (2.12)	18.2 (2.20)	<.001	.297	0.13 [−0.12, 0.39]
Sleep Tools	44.9 (1.12)	26.3 (1.23)	18.6 (1.28)	<.001	.161	0.15 [−0.03, 0.32]
**Total work impairment**						
Daily Boost	46.9 (2.08)	31.4 (2.28)	15.5 (2.36)	<.001		
Bedtime Stories	48.8 (2.02)	28.0 (2.27)	20.8 (2.34)	<.001	.109	0.21 [−0.05, 0.46]
Sleep Sounds	46.2 (2.02)	27.6 (2.18)	18.6 (2.27)	<.001	.338	0.12 [−0.13, 0.37]
Sleep Skills	45.5 (2.03)	26.6 (2.20)	18.6 (2.27)	<.001	.309	0.13 [−0.12, 0.38]
Sleep Tools	46.9 (1.17)	27.4 (1.28)	19.4 (1.32)	<.001	.146	0.15 [−0.02, 0.33]
**Activity impairment**						
Daily Boost	50.2 (2.11)	32.5 (2.28)	17.7 (2.39)	<.001		
Bedtime Stories	52.2 (2.06)	28.6 (2.29)	23.6 (2.40)	<.001	.081	0.22 [−0.03, 0.48]
Sleep Sounds	49.1 (2.03)	29.7 (2.18)	19.4 (2.29)	<.001	.601	0.07 [−0.18, 0.32]
Sleep Skills	48.0 (2.07)	27.7 (2.20)	20.4 (2.32)	<.001	.426	0.10 [−0.15, 0.36]
Sleep Tools	49.8 (1.18)	28.7 (1.28)	21.1 (1.35)	<.001	.217	0.13 [−0.05, 0.31]
**PSA—Cognitive**						
Daily Boost	24.6 (0.69)	19.0 (0.74)	5.65 (0.72)	<.001		
Bedtime Stories	25.1 (0.67)	20.3 (0.74)	4.83 (0.73)	<.001	.426	−0.10 [−0.35, 0.10]
Sleep Sounds	23.3 (0.66)	18.3 (0.71)	5.05 (0.69)	<.001	.547	−0.08 [−0.33, 0.17]
Sleep Skills	23.3 (0.68)	18.3 (0.71)	4.92 (0.70)	<.001	.471	−0.09 [−0.35, 0.16]
Sleep Tools	23.9 (0.39)	19.0 (0.74)	4.96 (0.41)	<.001	.404	−0.09 [−0.26, 0.09]
**PSA—Somatic**						
Daily Boost	14.9 (0.55)	13.4 (0.58)	1.55 (0.55)	.005		
Bedtime Stories	14.9 (0.53)	13.5 (0.58)	1.36 (0.55)	.014	.814	−0.03 [−0.28, 0.22]
Sleep Sounds	15.4 (0.53)	13.4 (0.56)	2.04 (0.53)	.001	.517	0.08 [−0.17, 0.33]
Sleep Skills	14.2 (0.54)	12.6 (0.56)	1.66 (0.53)	.002	.886	0.02 [−0.23, 0.27]
Sleep Tools	14.8 (0.31)	13.1 (0.33)	1.70 (0.31)	<.001	.806	0.03 [−0.15, 0.20]
**CERQ—Adaptive** ^*^						
Daily Boost	5.95 (0.13)	6.40 (0.14)	0.44 (0.13)	<.001		
Bedtime Stories	5.90 (0.13)	6.39 (0.14)	0.49 (0.13)	<.001	.801	−0.03 [−0.28, 0.22]
Sleep Sounds	5.86 (0.13)	6.04 (0.13)	0.19 (0.12)	.133	.155	0.18 [−0.07, 0.43]
Sleep Skills	6.09 (0.13)	6.52 (0.14)	0.43 (0.13)	.001	.961	0.01 [−0.25, 0.26]
Sleep Tools	5.95 (0.07)	6.31 (0.08)	0.37 (0.07)	<.001	.607	0.05 [−0.12, 0.23]
**CERQ—Maladaptive**						
Daily Boost	5.39 (0.13)	5.28 (0.14)	0.12 (0.13)	.377		
Bedtime Stories	5.63 (0.13)	5.28 (0.14)	0.35 (0.13)	.008	.205	0.16 [−0.28, 0.22]
Sleep Sounds	5.60 (0.13)	5.28 (0.14)	0.31 (0.13)	.012	.268	0.14 [−0.11, 0.39]
Sleep Skills	5.38 (0.13)	5.28 (0.14)	0.10 (0.13)	.427	.934	−0.01 [−0.26, 0.24]
Sleep Tools	5.54 (0.08)	5.28 (0.08)	0.26 (0.07)	<.001	.354	0.10 [−0.08, 0.27]

^*^Indicates variables where higher values reflect positive outcomes, whereas for all other variables, higher values indicate negative outcomes.

For the primary outcome measure, sleep disturbance, all within-group contrasts were significant, including a significant reduction in sleep disturbance in the control group (DB) (see Table 2). For between-group comparisons, standardized Hedges’ *g* effect sizes were very small and not statistically significant, with slightly greater reductions in sleep disturbance for the intervention groups than the control group. Finally, a very small, non-significant effect size was observed for the global effects of the combined Sleep Tools, favoring the intervention.

### Secondary outcomes

For changes in other questionnaire-based secondary outcomes, please refer to Table 2. For between-group comparisons on SRI-SF, very-small-to-small, non-significant effect sizes were observed for all Sleep Tools categories compared to the control group. Similarly, for the global combined efficacy of the Sleep Tools against the control, a small effect size was observed in favor of the intervention (*g* = 0.19, 95% CI [−0.01, 0.36]), which was non-significant (*p* = .077).

For self-reported sleep duration, participants in the control group reported an average increase of 33 min (se = 7 min) in sleep duration at Time Point 1 (t1) compared to Time Point 0 (t0). Participants in the BS group reported a 53-min increase in sleep duration (se = 7 min), with this difference being small, but statistically significant compared to the control group. Compared to the control group, non-significant differences in improved sleep duration were reported in the SS condition (average increase of 43 min, se = 7 min), nor in the SK condition (average increase of 25 min, se = 7 min). For the global effect of the combined Sleep Tools compared to the control condition, a very small, non-significant effect size was observed, favoring the intervention conditions.

For all other questionnaire outcomes, within-group effects from pre- to post-intervention were significant for all outcomes across all groups, except for the effects of SK on absenteeism, SS on adaptive cognitive emotion regulation, and DB and SK on maladaptive cognitive emotion regulation. However, between-group effects were very-small-to-negligible and all non-significant. This pattern remained consistent in the global efficacy of the sleep tools combined against the control condition.

The percentage of participants reporting a sleep onset latency exceeding 30 min at baseline decreased from decreased from 58.93% (*n* = 221) to 30.32% (*n* = 94) in the SS group, and from 60.51% (*n* = 72) to 33.67% (*n* = 33) in the control group (baseline to post-intervention) (see Table 3 for full results). A logistic regression revealed a significant effect of time (*b* = −0.78, *SE* = 0.20, OR = 0.46, 95% CI [0.31, 0.68], *p* < .001), suggesting that the odds of reporting a SOL of more than 30 min were 54% lower at post compared to pre-intervention. However, no significant Group × Time interactions were observed, indicating that this effect was consistent across all groups. Specifically, the Group × Time interaction was not significant for BS (*b* = −0.26, *SE* = 0.29, OR = 0.77, 95% CI [0.43, 1.40], *p* = .359), SS (*b* = −0.02, *SE* = 0.28, OR = 0.98, 95% CI [0.57, 1.67], *p* = .932), or SK (*b* = 0.10, *SE* = 0.28, OR = 1.10, 95% CI [0.63, 1.91], *p* = .726), suggesting that none of the intervention groups showed a significantly different change in sleep onset latency compared to the control group. No significant group difference was observed for the Sleep Tools combined compared to the control (*b* = −0.06, *SE* = 0.23, OR = 0.94, 95% CI [0.60, 1.49], *p* = .801).

**Table 3:** Self-Reported sleep onset latency for ITT analysis

Group	Time point	Self-reported sleep onset latency (*n*, %)				
		0–15 min	16–30 min	31–45 min	46–60 min	More than 60 min
Overall sample	t0 (*n* = 495)	52 (10.51)	150 (30.30)	116 (23.43)	98 (19.80)	79 (15.96)
	t1 (*n* = 408)	124 (30.39)	157 (38.48)	70 (17.16)	26 (6.37)	31 (7.60)
Daily Boost	t0 (*n* = 119)	10 (8.40)	37 (31.09)	28 (23.53)	23 (19.33)	21 (17.65)
	t1 (*n* = 98)	26 (26.53)	39 (39.80)	15 (15.31)	9 (9.18)	9 (9.18)
Bedtime Stories	t0 (*n* = 125)	11 (8.80)	35 (28.00)	35 (28.00)	25 (20.00)	19 (15.20)
	t1 (*n* = 96)	30 (31.25)	39 (40.62)	15 (15.62)	4 (4.17)	8 (8.33)
Sleep Sounds	t0 (*n* = 128)	15 (11.72)	35 (27.34)	31 (24.22)	27 (21.09)	20 (15.62)
	t1 (*n* = 108)	28 (25.93)	44 (40.74)	26 (24.07)	5 (4.63)	5 (4.63)
Sleep Skills	t0 (*n* = 123)	16 (13.01)	43 (34.96)	22 (17.89)	23 (18.70)	19 (15.45)
	t1 (*n* = 106)	40 (37.74)	35 (33.02)	14 (13.21)	8 (7.55)	9 (8.49)
Sleep Tools	t0 (*n* = 375)	42 (11.17)	113 (30.05)	88 (23.40)	75 (19.95)	58 (15.43)
	t1 (*n* = 310)	98 (31.61)	118 (38.06)	55 (17.74)	17 (5.48)	22 (7.10)

Finally, differences between groups on credibility and expect-ancy (measured using the CEQ [49] at baseline) were analyzed. One-way ANOVAs revealed significant differences across credibility scores across the four groups (*F* (3, 491) = 6.938, *p* < .001). Tukey’s HSD post-hoc test revealed no significant differences between the Sleep Tools, but significant differences were found between all experimental conditions and the control group. Specifically, the DB group had significantly lower credibility scores (*M* = 14.66, *SD* = 4.90) compared to the BS group (*M* = 17.01, *SD* = 4.43, *p* < .001), the SS group (*M* = 17.01, *SD* = 4.43, *p* < .001), and the SK group (*M* = 16.26, *SD* = 4.66, *p* = .041). Scores for expectancy were converted into *z*-scores. The DB group had a mean score of -0.21 (*SD* = 0.98), the BS group had a mean of 0.10 (*SD* = 1.07), the SS group reported a mean score of 0.07 (*SD* = 1.02), and the SK group had a mean of 0.03 (*SD* = 0.90). A one-way ANOVA was conducted, which revealed no statistically significant differences in CEQ Expectancy scores across the four groups (*F* (3, 491) = 2.44, *p* = .063).

### Objective engagement

The data for objective engagement are summarized in Table 4. Overall, 285 participants (75.80%) in the Sleep Tools conditions downloaded the MHapp and engaged with a sleep tool at least once, compared to 103 participants (86.55%) in the control group. A one-way ANOVA revealed a significant effect of group on ISO count (*F* (3, 384) = 4.78, *p* = .003). Post-hoc Tukey’s HSD test indicated that the DB group had a significantly higher mean ISO count compared to the BS group (mean difference = 3.84, 95% CI [0.03, 7.66], *p* = .048) and the SK group (mean difference = 5.29, 95% CI [1.50, 9.09], *p* = .002). No other group comparisons were statistically significant. Similarly, a one-way ANOVA showed a significant effect of group on ISC count (*F* (3, 384) = 4.13, *p* = .007). Tukey’s HSD test indicated that the DB group had a significantly higher ISC count than both the BS group (mean difference = 3.50, 95% CI [0.07, 6.92], *p* = .0432) and the SK group (mean difference = 4.36, 95% CI [0.95, 7.76], *p* = .006). No other pairwise comparisons were statistically significant.

**Table 4:** Summary of objective intervention engagement data for the study interventions and control condition

Variable	Experimental arms				Control
	Bedtime Stories (*n* = 125)	Sleep Sounds (*n* = 128)	Sleep Skills (*n* = 123)	Sleep Tool overall (*n* = 376)	Daily Boost (*n* = 119)
Number of people that downloaded and engaged with the Unmind app at least once (*n*, %)					
Mean (*SD*)	94 (75.20)	95 (71.88)	96 (78.05)	285 (75.80)	103 (86.55)
Individual sessions opened (ISO)					
Mean (*SD*)	10.15 (11.94)	11.80 (11.18)	8.70 (8.94)	10.21 (9.22)	13.99 (9.22)
Range	0–58	0–45	0–32	0–58	1–35
Individual sessions completed (ISC)					
Mean (*SD*)	8.32 (10.43)	9.0 (9.36)	7.46 (8.66)	8.26 (9.49)	11.82 (8.71)
Range	0–44	0–31	0–32	0–44	0–31
Sessions based on length (SBL) (in min)					
Mean (*SD*)	11.74 (16.62)	14.79 (20.36)	9.59 (11.83)	12.08 (16.78)	NA
Range	0–101	0–104	0–63	0–104	NA
Total playtime (TP) (in h)					
Mean (*SD*)	5.21 (8.22)	8.29 (12.54)	1.86 (2.69)	5.11 (9.14)	NA
Range	0–55.91	0–59.61	0–17.82	0–59.61	NA
Per protocol engagement					
(*n*, %)	29 (30.85)	32 (33.68)	26 (27.08)	87 (30.53)	54 (52.43)

Finally, engagement patterns by time of day were examined. To account for time zone differences and unconventional sleep–wake patterns, only UK participants were included, and shift workers were excluded. The analysis showed that 79.29% of DB usage occurred during daytime hours (6 am to 9 pm), while 83.57% of Sleep Tools usage took place during conventional sleep hours (9 pm to 6 am).

### Feedback questionnaire

Feedback questionnaire responses are shown in Table 5. Fisher’s exact test revealed significant differences in usability between the control and the Sleep Tools groups, with 75% of DB participants reporting immediate usability, compared to 46% in the Sleep Tools group (*p* < .001). Additionally, a significant difference was observed in participants’ likelihood to recommend the app feature, in favor of Sleep Tools (*p* = .029). No other significant differences were observed in the feedback questionnaire for relevance, negative effects, satisfaction, and quality, highlighting that participants overall rated and were satisfied with the allocated feature, regardless of whether they were in the control condition or not.

**Table 5:** Feedback questionnaire for individuals who downloaded the unmind app

Feedback question (*n*, %)	Experimental arms				Control	*p* value
	Bedtime Stories (*n* = 77)	Sleep Sounds (*n* = 85)	Sleep Skills (*n* = 84)	Sleep Tool overall (*n* = 246)	Daily Boost (*n* = 89)	
Did you find the Unmind app and intervention easy to use?						
No	0 (0.00)	1 (1.18)	(0.00)	1 (0.41)	(0.00)	<.001
Useable after a lot of time and effort	1 (1.30)	1 (1.18)	1 (1.19)	3 (1.22)	(0.00)	
Useable after some time and effort	7 (9.09)	9 (10.59)	9 (10.71)	25 (10.16)	2 (2.25)	
Easy to learn how to use	29 (37.66)	31 (36.47)	42 (50.00)	102 (41.46)	20 (22.47)	
Able to use app immediately	40 (51.95)	43 (50.59)	32 (38.1)	115 (46.75)	67 (75.28)	
Would you recommend this Unmind feature to people who might benefit from it?						
Not at all	1 (1.30)	2 (2.35)	2 (2.38)	5 (2.03)	4 (4.49)	.029^*^
Very few people	9 (11.69)	11 (12.94)	9 (10.71)	29 (11.79)	19 (21.35)	
Several people	31 (40.26)	29 (34.12)	34 (40.48)	94 (38.21)	25 (28.09)	
Many people	21 (27.27)	29 (34.12)	23 (27.38)	73 (29.67)	19 (21.23)	
Everyone	15 (19.48)	14 (16.47)	16 (19.05)	45 (18.29)	22 (24.72)	
Do agree that this Unmind feature felt relevant to your personal situation and needs?						
Strongly agree	14 (18.18)	13 (15.29)	17 (20.24)	44 (17.89)	15 (16.85)	.100
Agree	42 (54.55)	53 (62.35)	48 (57.14)	143 (58.13)	43 (48.31)	
Neither agree nor disagree	15 (19.48)	11(12.94)	10 (11.9)	36 (14.63)	15 (16.85)	
Disagree	3 (3.90)	8(9.41)	8 (9.52)	19 (7.72)	10 (11.24)	
Strongly disagree	3 (3.90)	0(0.00)	1 (1.19)	4 (1.63)	6 (6.74)	
Do you agree with this statement: “I experienced lasting bad effects from use of the Unmind Sleep Tools/Unmind Daily Boost”						
Strongly agree	0 (0.00)	0 (0.00)	0 (0.00)	0 (0.00)	0 (0.00)	.618
Agree	3 (3.90)	5 (5.88)	2 (2.38)	10 (4.07)	2 (2.25)	
I’m not sure	1 (1.30)	4 (4.71)	2 (2.38)	7 (2.85)	2 (2.25)	
Disagree	4 (5.19)	3 (3.53)	6 (7.14)	13 (5.28)	2 (2.25)	
Strongly disagree	69 (89.61)	73 (85.88)	74 (88.1)	216 (87.8)	83 (93.26)	
How satisfied are you with the Unmind feature you were asked to use?						
Very satisfied	19 (24.68)	19 (22.35)	21 (25.00)	59 (23.98)	22 (24.72)	.434
Satisfied	41 (53.25)	50 (58.82)	47 (55.95)	138 (56.10)	44 (49.44)	
Neither	14 (18.18)	10 (11.76)	7 (8.33)	31 (12.60)	14 (15.73)	
Dissatisfied	2 (2.60)	6 (7.06)	9 (10.71)	17 (6.91)	7 (7.87)	
Very Dissatisfied	1 (1.30)	0 (0.00)	0 (0.00)	1 (0.41)	2 (2.25)	
How would you rate the quality of the Unmind feature you were asked to use?						
Poor	1 (1.30)	1 (1.18)	1 (1.19)	3 (1.22)	2 (2.25)	.162
Okay	12 (15.58)	13 (15.29)	16 (19.05)	41 (16.67)	23 (25.84)	
Good	39 (50.63)	46 (54.12)	38 (45.24)	123 (50.00)	36 (40.45)	
Excellent	25 (32.47)	25 (29.41)	29 (34.51)	79 (32.11)	28 (31.46)	

A Fisher’s exact test was used to examine the association between responses to the feedback questions for sleep tools overall and the control group (Daily Boost).

^*^indicates differences in outcomes at the p < 0.05 level of signficance

### Negative effects

Twelve participants (3.58% of participants who downloaded the app) self-reported negative effects from the study (see Table 5). These participants were asked to describe lasting negative effects they experienced from using their allocated MHapp feature. Among the reported negative effects were reliance on the app to get to sleep when it wasn’t necessary (BS), difficulty navigating the app (SS), ear itching (SS), and waking up due to thinking about the content (SK). When asked to describe what about the use of their allocated app feature, they thought led to the negative effects, some participants mentioned that the process was too time-consuming or made them more stressed than before (SS), while others cited loud volume (SS) or boredom (SK). A verbatim output of these participant responses can be found in supplementary material (Supplementary Material Measure S3). Meaningful deterioration in the primary outcome measure of self-reported sleep disturbance was analyzed using the Reliable Change Index (RCI) [52]. A reliable deterioration was defined as a change greater than 5.4, the most conservative cutoff based on previous research [53]. Among the 408 participants who completed the follow-up questionnaire, 10 individuals (2.45%) showed a meaningful deterioration in their sleep disturbance. This included 1 participant (1.02%) in the control group, 3 (3.13%) in the BS group, 1 (0.93%) in the SS group, and 5 (4.71%) in the SK group. In terms of mental health symptoms, no participants showed a meaningful RCI score [52] on the PHQ-4, indicating that none reported a significant deterioration in mental health during the study period.

### P‌P analysis

As shown in Table 4, the PP analysis included 141 participants, who engaged with their assigned feature at least 12 times during the study, with an average engagement of 30.53% for the sleep tools groups and 52.43% for the control group, indicating greater engagement rates in the control condition.

One-way ANOVAs were conducted to investigate whether baseline differences existed between those included in the PP analysis and those excluded. No significant differences were found. For raw, completed cases and the LME model results from the PP analysis, see Tables S3 and S4, respectively. Regarding the primary outcome measure, sleep disturbance, all within-group contrasts were statistically significant, indicating reductions in sleep disturbance from pre- to post-intervention across all groups. Between-group comparisons, assessed using standardized Hedges’ *g* effect sizes, showed a medium-sized negative effect in favor of the control group for BS, a very small, non-significant negative effect for SS, and a small, non-significant negative effect for SK. For Sleep Tools combined, a small to medium, non-significant negative effect size favoring the control group was observed.

### Exploratory analyses

Planned exploratory analyses examined changes in cognitive and somatic pre-sleep arousal across conditions. As shown in Table 4, ITT analyses revealed significant pre-post reductions in both arousal types across all groups, including the control, with very small and non-significant between-group effect sizes.

For adaptive cognitive emotion regulation, all groups except SS showed pre-post increases, but between-group differences were small and non-significant. Maladaptive emotion regulation significantly decreased for BS and SS, but not for SK, with similarly small and non-significant between-group effects (see Table 4). For PP analysis results, see Table S4 (Supplementary Material).

To further understand the primary outcome findings, several unplanned exploratory analyses were conducted. A sensitivity analysis using multiple imputation (30 datasets, predictive mean matching) was performed to address missing follow-up data due to dropout. This analysis revealed no differences in the magnitude, direction, or statistical significance in findings compared to the ITT analysis across LMEs, indicating the robustness of the primary findings. Similarly, a further exploratory analysis was conducted, including only participants who downloaded the app and engaged with their allocated feature at least once. The results were consistent with the ITT analysis. Additionally, a moderated regression analysis was conducted to assess whether baseline sleep disturbance influenced changes in sleep disturbance over time. The analysis indicated that higher baseline scores were associated with greater improvements from pre- to post-intervention across all groups (*b* = 0.497, *SE* = 0.118, *t*(400) = −4.201, *p* < .001). However, no intervention group (BS, SS, SK) showed a statistically significant main effect on change in sleep disturbance relative to the control group, and the interaction terms were not significant. Finally, exploratory analyses examined the relationship between objective engagement and changes in self-reported sleep disturbance. Linear regression models did not show a statistically significant effect (*b* = 0.070, *SE* = 0.050, *t* = 1.40, *p* = .161), suggesting no dose–response relationship.

## Discussion

The present study was the largest RCT to date to examine the effectiveness of three categories of standalone, audio-based interventions (BS, SS, and SK) compared to an active digital control condition (the DB) in a population of working adults with self-reported sleep disturbance. Findings did not support the primary hypothesis that Sleep Tools would be more effective than the control condition in reducing self-reported sleep disturbance post-intervention. Very small, non-significant effects in favor of the intervention were observed for both self-reported sleep disturbance and sleep-related impairment, and secondary hypotheses regarding mental health symptoms, well-being, and work-related impairment were not supported. Between-group differences in sleep duration were non-significant, except for BS, which showed a significant medium-sized effect and a greater increase in sleep duration compared to the control condition. One possibility is that these sleep tools may confer specific benefit for sleep duration, which warrants further investigation. Although within-group improvements were observed across most outcomes, these findings indicate that audio-based sleep tools were not more effective than a digital control condition, and improvement associated with the use of MHapp-delivered Sleep Tools cannot be assumed to reflect their active, sleep-specific ingredients. Finally, exploratory analyses assessing potential mechanisms did not indicate a significantly greater effect of the Sleep Tools compared to the control condition on either pre-sleep arousal or cognitive emotion regulation.

### Findings in context

The findings of this study are unexpected within the sleep app space, given the widespread use of sleep tools and evidence suggesting that their use is associated with improved sleep disturbance [30]. The absence of evidence presented here suggests that previous app-based studies with less robust research designs may be overestimating the specific effects of these tools [21, 30, 32]. This may, at least in part, be attributable to differences in inactive versus active control conditions and their influence on effect sizes, as observed in other mental health app research [28]. In the current study, the active control condition was matched for user experience, engagement frequency, and expectancy (despite lower credibility), raising the possibility that observed sleep improvements may have been driven by MHapp engagement rather than sleep-specific components.

### Strengths

This study is the first to evaluate the efficacy of app-delivered, standalone, audio-based interventions for sleep against a well-matched digital control condition, rather than a waitlist control, making it one of the most rigorous evaluations of audio-based sleep tools to date. Conducted in a naturalistic setting, the study employed a real-world app that participants independently downloaded and used at home, reflecting typical engagement with this type of content. It featured a large, decentralized sample with a balanced gender distribution and diverse representation across occupations. The digital control condition, drawn from existing app content, was designed to match the intervention for user experience and engagement frequency, while withholding the presumed active ingredient, the Sleep Tools. No significant differences in expectancy were observed between conditions, allowing for control of non-specific effects related to expectations associated with engaging with a MHapp. Furthermore, objective engagement metrics indicated that the control condition was well matched for user interest, even outperforming the intervention. Time-of-day usage analyses confirmed that participants primarily used the control condition during the daytime, and the Sleep Tools at night, ruling out the possibility that the control condition content (the “Daily Boost”) was used as a Sleep Tool. Engagement was objectively tracked, enabling us to assess intervention adherence and perform PP analysis. Moreover, study retention was considerably higher (82.42%) than the average attrition rates observed in digital mental health interventions [54]. Finally, sensitivity analyses with imputed data revealed no significant differences compared to the ITT model, suggesting that findings were not influenced by dropout status, further reinforcing the robustness of the results.

### Limitations, methodological considerations, and future research

The study also has several limitations. Firstly, the intervention may have been insufficiently potent. PP engagement rates were lower (BS: 30.85%; SS: 33.68%; SK: 27.08%) than those reported for technology-mediated insomnia treatments (estimated at ~52%) [55], suggesting participants may not have engaged sufficiently with the intervention. Moreover, although the Sleep Tools content used in the current study appears broadly similar to that of other widely used apps [30, 31], it is possible that specific active ingredients available from sleep tools of this kind were inadvertently omitted here. For example, the SK tracks were delivered as voice-narrated audio only, whereas other apps often combine such techniques with music or soundscapes, which could influence efficacy. Additionally, the platform used in this study is a general MHapp, and its sleep tools library may be less extensive than dedicated sleep apps, potentially limiting personalization and alignment with user preferences [56]. However, this is difficult to determine, as little is known about the specific characteristics of audio content that drive user preference and potential effects, and future research should priorities identifying these components, rather than focusing on single products [57]. It is also possible that greater use frequency, duration, or overall study length is needed to produce measurable effects; however, no dose–response relationship was observed in sensitivity analyses or in the PP analysis. Future research should examine more prescriptive engagement protocols, or longer study durations would bring stronger effects.

Secondly, allocating participants to a single sleep tools category may have been at odds with individual preferences or the flexible needs-matched use of these tools. Pilot findings suggest that participants tend to favor one category of tools over the other, given the option [21,], and enjoyment and personal preference may influence efficacy, as proposed in research on the effects of music on sleep [58]. Future research should evaluate these tools as a broad menu of options, allowing free choice over content to improve engagement and better reflect real-world use.

Thirdly, the current sample may not have been best suited to benefit from Sleep Tools. The relatively liberal inclusion criterion for sleep disturbance (JSS ≥ 7) may have diluted a genuine treatment effect by including participants with mild sleep disturbance who were unlikely to benefit from intervention. Although higher baseline sleep disturbance was associated with greater improvement, this was not specific to the intervention groups suggesting no specific advantage of these tools. Additionally, participants were relatively young and highly educated compared to clinical insomnia samples. Moreover, 69.49% of the sample scored above the PHQ-4 cutoff (≥3), indicating clinically relevant anxiety and/or depression, substantially higher than the estimated 25% in the general population [51]. The elevated rate of mental health symptoms may limit the generalizability of findings and highlight the need for future research to investigate which populations are most likely to benefit from audio-based sleep interventions.

Fourthly, although sleep disturbance was assessed using a validated scale, quantitative metrics of sleep (such as total sleep duration and sleep onset latency) were measured using single-item self-reports, which may be unreliable [59]. The study did not include objective sleep measures, which is an important limitation given the known discrepancies between subjective and objective sleep metrics [60], especially in individuals with insomnia [61]. Future studies should incorporate objective measures of sleep, such as actigraphy, to better understand whether observed improvements reflect genuine physiological changes in sleep. This is especially relevant given emerging evidence that certain auditory sleep interventions (pink noise) may have complex and potentially adverse effects on sleep architecture [62].

Fifthly, the MHapp-based digital control condition was not beta tested, and although it was not designed to support sleep, we cannot rule out the possibility that aspects of the control condition may have influenced sleep-related outcomes indirectly. This highlights the need for future research to develop and test well-matched control conditions that, as recommended [22], match the intervention experience in as many ways as possible. Doing so will support the standardization of research methods and improve the replicability of findings in future trials.

Finally, although the study was powered based on an a priori sample size calculation, the original calculation was based on individual pairwise comparisons and did not adjust for the presence of multiple intervention arms, which may have reduced statistical power for each contrast. This is particularly relevant given that the observed between-group effects were in the expected direction, albeit very small. Future research should determine whether effects of this magnitude are meaningful at a population level, especially considering the scalability, popularity, and safety of these tools, and whether trials sufficiently powered to detect such subtle differences are justified.

### Practical implications

The findings do not support a specific recommendation of Sleep Tools for individuals with self-reported sleep disturbance, given the lack of significant superiority over the control condition. Nevertheless, their use is not discouraged, particularly if people find them enjoyable or incorporate them into a wind-down routine. These tools were generally safe, with no meaningful deterioration in mental health observed and only a small proportion of participants (2.45%) showing a meaningful worsening of sleep disturbance during the study period. Regardless, individuals experiencing significant or persistent sleep problems should seek professional, evidence-based care.

An additional consideration is the app-based delivery of interventions, which involves phone use at bedtime. Although traditional sleep hygiene recommendation advice against bedtime screen use [63], and some evidence supports this view [64, 65], the present study suggests a more complex picture as sleep outcomes improved across all groups. Structured, purposeful engagement with MHapp-based content may not be inherently detrimental to sleep and could represent a less disruptive alternative to other forms of bedtime phone use, consistent with emerging perspectives on technology and sleep [66, 67]. Finally, the within-group improvements observed across all conditions suggest that MHapps may play a broader role in supporting sleep and well-being, even if partly driven by expectancy effects. Such effects should be more systematically measured and considered in the design and evaluation of digital health interventions.

## Conclusions

This large RCT found that standalone, audio-based sleep interventions delivered via a MHapp over 4 weeks did not lead to superior improvements in sleep disturbance, mental health, or well-being compared to a well-matched digital control condition. Between-group effects were very small and in the expected direction, and these findings do not support the use of Sleep Tools as a standalone intervention for individuals with sleep disturbance. Future research should examine these tools when offered as a broad menu of options, incorporate objective sleep measures and determine whether effects of this magnitude are meaningful at a population level.

## Supplementary Material

USleep_Supplementary_Material_zsag154

## Data Availability

In line with UKRI’s open research data policy, the data generated and analyzed during the current study is available from the corresponding author on request.
