# Supplementary material for: USleep: efficacy of app-based audio interventions to improve sleep disturbance in working adults, a multi-arm randomized controlled trial
Source: Sleep. 2026 Jun 1;49(9):zsag154. doi: 10.1093/sleep/zsag154 (PMC13553283; doi:10.1093/sleep/zsag154)
Supplement: Supplementary file 1 [file usleep_supplementary_material_zsag154.docx]

**Title: USleep: Efficacy of App-Based Audio Interventions to Improve Sleep Disturbance in Working Adults, a Multi-Arm Randomised Controlled Trial**

**Authors and authors’ affiliations:**

**Jessica Vazzaz^1*^, Faith Matcham^1^, Marcos Economides^2^, Heather Bolton ^2^, Kate Cavanagh^1^**

**^1^ School of Psychology, University of Sussex, East Sussex, UK**

**^2^ Science Department, Unmind Ltd, London, UK**

*** Corresponding author: Jessica Vazzaz, School of Psychology, University of Sussex,** Falmer, Brighton, BN1 9RH, United Kingdom. Email address: [j.vazzaz@sussex.ac.uk](mailto:j.vazzaz@sussex.ac.uk)

**Supplementary Material Capture List:**

**Measure S1.** Feedback Questionnaire (intervention groups)

**Measure S2.** Feedback Questionnaire (control group)

**Table S1.** Raw, complete case scores at *t0* and t1 divided by county.

**Table S2.** Raw, complete case scores at *t0* and *t1* for all groups for ITT

**Measure S3. Open text answer to feedback questions:**

**Table S3.** Raw, complete case scores at *t0* and *t1* for all groups for PP Analysis.

**Table S4.** Estimated marginal means (PP Sample) from linear mixed effect models with standardised effect sizes.

**Measure S1.** Feedback Questionnaire (intervention groups)

Please answer the questions in this section as honestly as possible - your answers won’t affect the payment you receive for taking part.

Part 1: The following questions will explore whether and how you used the Sleep Tools you were invited to use, and the factors that may have influenced how often you used them.

1. Did you successfully sign up to the Unmind platform as part of this study?
   a. Yes
   b. No

→ If No, exit the feedback questionnaire after the following question:

Please indicate your reasons for not signing up to Unmind, selecting all that apply:

- I wasn’t able to create an Unmind account, or the instructions were unclear
- I didn’t realise that I was supposed to sign up to Unmind
- I forgot that I was taking part in the study
- I changed my mind about taking part in the study
- I experienced technical difficulties that I wasn’t able to resolve
- I didn’t have access to the Internet
- I had a change in circumstance that prevented me from taking part in the study (such as falling ill)
- Other (optional comment)

2. On average, how often do you think you used the Unmind Sleep Tools you were asked to use over the last 4 weeks?
   a. I didn’t use them at all
   b. Once or twice a week
   c. At least 3 times a week
   d. Most nights of the week
   e. Every night

→ If ‘a’ is chosen, exit the feedback questionnaire after this question: Please indicate your reasons for not using the Unmind Sleep Tools, selecting all that apply. Try to be as honest as you can.

- I didn’t have enough time to use the Sleep Tools
- I forgot that I was taking part in the study, or that I was supposed to use them
- I lost motivation to take part in the study
- I didn’t feel like the Sleep Tools would help with anything
- I experienced technical difficulties that prevented me from using them
- I didn’t have access to a smartphone or to the Internet
- I had a change in circumstance that prevented me from using them (such as falling ill)
- Other (optional comment)

→ If ‘a’ is *not* chosen: Please indicate your reasons for not using the Unmind Sleep Tools more often, selecting all that apply. Try to be as honest as you can.

If you used them every night, try to think of any reasons that might prevent you from using them going forwards, or choose ‘Other’ instead.

- I didn’t have enough time to use the Sleep Tools
- I lost motivation to take part in the study
- I didn’t feel like the Sleep Tools were helping
- I didn’t enjoy using the Sleep Tools
- I experienced technical difficulties that prevented me from using them
- I had a change in circumstance that prevented me from using them more often (such as falling ill)
- I used other Tools within the Unmind platform to help with my sleep instead
- My sleep naturally improved, so I didn’t feel I needed to use them as much
- Other (optional comment)

Part 2: The following questions will ask about your opinions and satisfaction with the Sleep Tools you were asked to use.

3. Did you find the Unmind app and Sleep Tools easy to use?
   a. No (limited instructions; confusing menu labels/icons; complicated)
   b. Useable after a lot of time and effort
   c. Useable after some time and effort
   d. Easy to learn how to use the app (or has clear instructions)
   e. Able to use app immediately; intuitive; simple
4. Would you recommend the Sleep Tools you were asked to use to people who might benefit from them, such as friends, family, or colleagues?
   a. Not at all – I would not recommend them to anyone
   b. There are very few people I would recommend them to
   c. Maybe – There are several people whom I would recommend them to
   d. There are many people I would recommend them to
   e. Definitely – I would recommend them to everyone
5. Would you agree that the Unmind Sleep Tools felt relevant to your personal situation and needs?
   a. Strongly agree
   b. Agree
   c. Neither agree nor disagree
   d. Disagree
   e. Strongly disagree
6. Please indicate whether you agree or disagree with the following statement: “I experienced lasting bad effects from use of the Unmind Sleep Tools”
   a. Strongly agree
   b. Slightly agree
   c. I’m not sure
   d. Slightly disagree
   e. Strongly disagree

→ If ‘a’ or ‘b’ is selected: Please describe 1) the lasting bad effect, and 2) what it was about the use of the Sleep Tools you think led to the lasting bad effect.

7. How satisfied are you with the Unmind Sleep Tools you were asked to use overall?
   a. Very satisfied
   b. Satisfied
   c. Neither satisfied nor dissatisfied
   d. Dissatisfied
   e. Very dissatisfied
8. How would you rate the quality of the Unmind Sleep Tools you were asked to use?
   a. Excellent
   b. Good
   c. Okay
   d. Poor
9. Do you have any other feedback on the Unmind Sleep Tools (or the Unmind app in general), including suggestions for improvement? (Free text/comment box)

**Measure S2**. Feedback Questionnaire (control group)

Please answer the questions in this section as honestly as possible - your answers won’t affect the payment you receive for taking part.

Part 1: The following questions will explore whether and how you engaged with the Unmind feature you were invited to use, and the factors that may have influenced how often you used them.

1. Did you successfully sign up to the Unmind platform as part of this study?
   a. Yes
   b. No

→ If No, exit the feedback questionnaire after the following question:

Please indicate your reasons for not signing up to Unmind, selecting all that apply:

- I wasn’t able to create an Unmind account, or the instructions were unclear
- I didn’t realise that I was supposed to sign up to Unmind
- I forgot that I was taking part in the study
- I changed my mind about taking part in the study
- I experienced technical difficulties that I wasn’t able to resolve
- I didn’t have access to the Internet
- I had a change in circumstance that prevented me from taking part in the study (such as falling ill)
- Other (optional comment)

2. On average, how often do you think you engaged with the Unmind Daily Boost over the last 4 weeks?
   a. I didn’t engage with them at all
   b. Once or twice a week
   c. At least 3 times a week
   d. Most days of the week
   e. Every day

→ If ‘a’ is chosen, exit the feedback questionnaire after this question: Please indicate your reasons for not using the Unmind Daily Boost, selecting all that apply. Try to be as honest as you can.

- I didn’t have enough time to engage with the Daily Boost
- I forgot that I was taking part in the study, or that I was supposed to use them
- I lost motivation to take part in the study
- I didn’t feel like the Daily Boost would help with anything
- I experienced technical difficulties that prevented me from using them
- I didn’t have access to a smartphone or to the Internet
- I had a change in circumstance that prevented me from using them (such as falling ill)
- Other (optional comment)

→ If ‘a’ is *not* chosen: Please indicate your reasons for not engaging with the Unmind Daily Boost more often, selecting all that apply. Try to be as honest as you can.

If you used them every day, try to think of any reasons that might prevent you from using them going forwards, or choose ‘Other’ instead.

- I didn’t have enough time to engage with the Daily Boost
- I lost motivation to take part in the study
- I didn’t feel like the Daily Boosts were helping
- I didn’t enjoy using the Daily Boost
- I experienced technical difficulties that prevented me from using them
- I had a change in circumstance that prevented me from using them more often (such as falling ill)
- I used other features of the Unmind platform instead
- Other (optional comment

Part 2: The following questions will ask about your opinions and satisfaction with the Unmind feature you were asked to use.

3. Did you find the Unmind app and Daily Boost feature easy to use?
   a. No (limited instructions; confusing menu labels/icons; complicated)
   b. Useable after a lot of time and effort
   c. Useable after some time and effort
   d. Easy to learn how to use the app (or has clear instructions)
   e. Able to use app immediately; intuitive; simple
4. Would you recommend the Unmind Daily Boost feature to people who might benefit from them, such as friends, family, or colleagues?
   a. Not at all – I would not recommend them to anyone
   b. There are very few people I would recommend them to
   c. Maybe – There are several people whom I would recommend them to
   d. There are many people I would recommend them to
   e. Definitely – I would recommend them to everyone
5. Would you agree that the Unmind Daily Boosts felt relevant to your personal situation and needs?
   a. Strongly agree
   b. Agree
   c. Neither agree nor disagree
   d. Disagree
   e. Strongly disagree
6. Please indicate whether you agree or disagree with the following statement: “I experienced lasting bad effects from use of the Unmind Daily Boost”
   a. Strongly agree
   b. Slightly agree
   c. I’m not sure
   d. Slightly disagree
   e. Strongly disagree

→ If ‘a’ or ‘b’ is selected: Please describe 1) the lasting bad effect, and 2) what it was about the use of the Daily Boost you think led to the lasting bad effect.

7. How satisfied are you with the Unmind feature (Daily Boost) that you were asked to use overall?
   a. Very satisfied
   b. Satisfied
   c. Neither satisfied nor dissatisfied
   d. Dissatisfied
   e. Very dissatisfied
8. How would you rate the quality of the Unmind Daily Boost you were asked to use?
   a. Excellent
   b. Good
   c. Okay
   d. Poor
9. Do you have any other feedback on the Unmind Daily Boost (or the Unmind app in general), including suggestions for improvement? (Free text/comment box)

**Table S1.** Raw, complete case scores at *t0* and t1 divided by county.

| Outcome | Estimates (mean, SE) | |
| --- | --- | --- |
|  | *t0* | *t1* |
| **SD-SF** |  |  |
| UK | 57.95 (0.38) | 49.41 (0.50) |
| US | 57.04 (0.55) | 48.74 (0.78) |
| **SRI-SF** |  |  |
| UK | 61.15 (0.43) | 52.24 (0.58) |
| US | 60.51 (0.59) | 51.80 (0.89) |
| **Sleep duration** |  |  |
| UK | 6.23 (0.07) | 6.92 (0.06) |
| US | 6.25 (0.11) | 6.71 (0.11) |
| **PHQ-4** |  |  |
| UK | 4.51(0.18) | 3.04 (0.18) |
| US | 4.91 (0.26) | 3.30 (0.28) |
| **SWEMWBS** |  |  |
| UK | 20.22 (0.20) | 22.11 (0.27) |
| US | 20.62 (0.31) | 22.40 (0.39) |
| **WPAI** |  |  |
| *Absenteeism* |  |  |
| UK | 4.42 (0.51) | 1.67 (0.30) |
| US | 5.55 (0.84) | 3.80 (0.78) |
| *Presenteeism* |  |  |
| UK | 45.51 (1.16) | 27.75 (1.35) |
| US | 43.56 (1.68) | 24.93 (2.01) |
| *Total work impairment* |  |  |
| UK | 47.48 (1.20) | 28.67 (1.38) |
| US | 45.75 (1.72) | 26.62 (2.16) |
| *Activity impairment* |  |  |
| UK | 49.9 (1.27) | 29.58 (1.42) |
| US | 49.9 (1.63) | 28.69 (2.10) |
| **PSA Cognitive** |  |  |
| UK | 38.86 (0.7) | 32.10 (0.72) |
| US | 39.08 (0.93) | 31.79 (0.98) |
| **PSA Somatic** |  |  |
| UK | 14.79 (0.35) | 13.05 (0.33) |
| US | 15.01 (0.47) | 13.18 (0.47) |
| **CERQ Adaptive** |  |  |
| UK | 5.84 (0.08) | 6.31 (0.09) |
| US | 6.11 (0.10) | 6.38 (0.12) |
| **CERQ Maladaptive** |  |  |
| UK | 5.5 (0.08) | 5.25 (0.09) |
| US | 5.5 (0.11) | 5.28 (0.12) |

SE = standard error of the estimated marginal means (EMM) from the ITT sample; t0 = baseline, t1 = post-intervention (4 weeks). Abbreviations: SD-SF = PROMIS Sleep Disturbance Short Form; SRI-SF = PROMIS Sleep-Related Impairment Short Form; PHQ-4 = Patient Health Questionnaire; SWEMWBS = Short Warwick-Edinburgh Mental Wellbeing Scale; WPAI = Work Productivity and Activity Impairment; PSA = Pre-Sleep Arousal; CERQ = Cognitive Emotion Regulation Questionnaire.

**Table S2**. Raw, complete case scores at *t0* and *t1* for all groups for ITT.

| Outcome | Estimates (mean, SE) | |
| --- | --- | --- |
|  | *t0* | *t1* |
| **SD-SF** |  |  |
| Overall Sample | 57.60 (0.32) | 49.17 (0.42) |
| Daily Boost | 58.07 (0.60) | 50.37 (0.76) |
| Bedtime stories | 57.46(0.60) | 48.51 (0.90) |
| Sleep sounds | 57.53 (0.67) | 49.10 (0.91) |
| Sleep Skills | 57.36 (0.68) | 49.12 (0.91) |
| **SRI-SF** |  |  |
| Overall Sample | 60.90 (0.35) | 52.08 (0.49) |
| Daily Boost | 60.86 (0.65) | 53.27 (0.99) |
| Bedtime stories | 60.99 (0.71) | 51.22 (1.06) |
| Sleep sounds | 60.87 (0.74) | 52.48 (0.90) |
| Sleep Skills | 60.88 (0.67) | 51.36 (0.95) |
| **Sleep duration** |  |  |
| Overall Sample | 6.24 (0.06) | 6.58 (0.06) |
| Daily Boost | 6.08 (0.10) | 6.62 (0.12) |
| Bedtime stories | 6.12 (0.12) | 6.93 (0.11) |
| Sleep sounds | 6.27 (0.10) | 6.98 (0.11) |
| Sleep Skills | 6.46 (0.13) | 6.83 (0.11) |
| **PHQ-4** |  |  |
| Overall Sample | 4.67 (0.15) | 3.13 (0.15) |
| Daily Boost | 4.45 (0.31) | 3.07 (0.32) |
| Bedtime stories | 4.86 (0.28) | 2.81 (0.29) |
| Sleep sounds | 4.94 (0.31) | 3.26 (0.31) |
| Sleep Skills | 4.39 (0.29) | 3.35 (0.29) |
| **SWEMWBS** |  |  |
| Overall Sample | 20.37 (0.17) | 22.21 (0.22) |
| Daily Boost | 20.31 (0.32) | 21.88 (0.45) |
| Bedtime stories | 20.86 (0.36) | 23.31 (0.51) |
| Sleep sounds | 19.85 (0.35) | 22.01 (0.41) |
| Sleep Skills | 20.48 (0.35) | 21.73 (0.40) |
| **WPAI** |  |  |
| *Absenteeism* |  |  |
| Overall Sample | 4.86 (0.45) | 2.42 (0.34) |
| Daily Boost | 5.93 (1.22) | 3.30 (0.81) |
| Bedtime stories | 5.27 (0.76) | 1.89 (0.51) |
| Sleep sounds | 4.17(0.87) | 2.15 (0.72) |
| Sleep Skills | 4.11 (0.68) | 2.36 (0.64) |
| *Presenteeism* |  |  |
| Overall Sample | 44.75 (0.96) | 26.75 (1.12) |
| Daily Boost | 44.40(2.01) | 29.13 (2.21) |
| Bedtime stories | 46.50 (1.92) | 27.07 (2.54) |
| Sleep sounds | 44.43(1.90) | 26.04 (2.04) |
| Sleep Skills | 43.64 (1.88) | 25 (2.22) |
| *Total work impairment* |  |  |
| Overall Sample | 46.8 (0.99) | 27.95 (1.17) |
| Daily Boost | 46.64 (2.05) | 30.77 (2.33) |
| Bedtime stories | 48.83 (1.99) | 27.96 (2.60) |
| Sleep sounds | 46.22 (1.99) | 27.17 (2.16) |
| *Activity impairment* |  |  |
| Overall Sample | 49.9 (1.00) | 29.26 (1.18) |
| Daily Boost | 50.17(1.99) | 31.53 (2.43) |
| Bedtime stories | 52.24 (1.87) | 28.44 (2.47) |
| Sleep sounds | 49.14 (2.03) | 29.26 (2.20) |
| Sleep Skills | 48.05(2.12) | 27.92 (2.38) |
| **PSA Cognitive** |  |  |
| Overall Sample | 24.07 (0.34) | 18.89 (0.37) |
| Daily Boost | 24.62 (0.63) | 19.03 (0.72) |
| Bedtime stories | 25.11 (0.66) | 19.99 (0.78) |
| Sleep sounds | 23.33 (0.74) | 18.26 (0.74) |
| Sleep Skills | 23.27 (0.68) | 18.41 (0.70) |
| **PSA Somatic** |  |  |
| Overall Sample | 14.87 (0.28) | 13.1 (0.27) |
| Daily Boost | 14.95 (0.54) | 13.33 (0.55) |
| Bedtime stories | 14.86 (0.57) | 13.23 (0.56) |
| Sleep sounds | 15.41 (0.60) | 13.32 (0.54) |
| Sleep Skills | 14.24 (0.54) | 12.53 (0.50) |
| **CERQ Adaptive** |  |  |
| Overall Sample | 5.95 (0.06) | 6.34 (0.07) |
| Daily Boost | 5.95 (0.13) | 6.36 (0.15) |
| Bedtime stories | 5.90 (0.14) | 6.43 (0.15) |
| Sleep sounds | 5.86 (0.12) | 6.06 (0.14) |
| Sleep Skills | 6.09 (0.12) | 6.51 (0.13) |
| **CERQ Maladaptive** |  |  |
| Overall Sample | 5.5 (0.07) | 5.26 (0.07) |
| Daily Boost | 5.39 (0.14) | 5.24 (0.16) |
| Bedtime stories | 5.63 (0.13) | 5.23 (0.13) |
| Sleep sounds | 5.60 (0.14) | 5.28 (0.15) |
| Sleep Skills | 5.38 (0.12) | 5.29 (0.13) |

SE = standard error of the estimated marginal means (EMM) from the ITT sample; t0 = baseline, t1 = post-intervention (4 weeks). Abbreviations: SD-SF = PROMIS Sleep Disturbance Short Form; SRI-SF = PROMIS Sleep-Related Impairment Short Form; PHQ-4 = Patient Health Questionnaire; SWEMWBS = Short Warwick-Edinburgh Mental Wellbeing Scale; WPAI = Work Productivity and Activity Impairment; PSA = Pre-Sleep Arousal; CERQ = Cognitive Emotion Regulation Questionnaire.

**Measure S3. Open text answer to feedback questions:**

**Please describe the lasting bad effect(s) you experienced from use of the Unmind Sleep Tools:**

**Bedtime Stories:**

- "Reliance on using the app to get to sleep when I didn't need to. Became habitual."
- "I still have occasional bad dreams that wake me up."
- "There weren’t really any bad effects, just at the start it was a different thing to get used to."

**Sleep Sounds:**

- "Nothing really."
- "I felt that the app itself was difficult to log into and navigate, and was a lot of hassle."
- "I didn’t experience any bad effects."
- "Ear itching."
- "Bad effects."

**Sleep Skills:**

- "I seemed unmotivated."
- "It didn’t help much, but it did provide useful information that helped me understand my sleep habits."
- "Woke me up due to thinking about the contents."

**Daily Boost:**

- "App very buggy."
- "Just having technical issues on the app."

**Please describe what it was about the use of the Sleep Tools/Daily Boost you think led to the last bad effect:**

**Bedtime Stories:**

- "Repetition of use as I was doing it to form a response to a study rather than using it when I needed to."
- "No idea."
- "Something new that I don’t really do."

**Sleep Sounds:**

- "No bad effect."
- "I think that the whole process was too time consuming and it made me more stressed than before."
- "Nothing."
- "Loud volume."
- "No bad effects."

**Sleep Skills:**

- "I was bored of the same routine."
- "It didn’t have a bad effect."
- "Same reason as above."

**Daily Boost:**

- "Easy to use when working properly."
- "I like the positivity it was trying to give you daily."

**Table S3**. Raw, complete case scores at *t0* and *t1* for all groups for PP Analysis.

| Outcome | Estimates (mean, SE) | |
| --- | --- | --- |
|  | *t0* | *t1* |
| **SD-SF** |  |  |
| Overall Sample | 57.68 (0.62) | 49.23 (0.69) |
| Daily Boost | 59.64 (0.94) | 49.37 (1.05) |
| Bedtime stories | 55.74 (1.02) | 50.67 (1.36) |
| Sleep sounds | 57.98 (1.39) | 48.91 (1.58) |
| Sleep Skills | 55.76 (1.64) | 47.79 (1.70) |
| **SRI-SF** |  |  |
| Overall Sample | 60.70 (0.66) | 51.61 (0.82) |
| Daily Boost | 61.00 (1.15) | 51.22 (1.42) |
| Bedtime stories | 60.67 (1.46) | 53.31 (1.88) |
| Sleep sounds | 61.00 (1.44) | 52.07 (1.69) |
| Sleep Skills | 59.78 (1.33) | 49.90 (1.67) |
| **Sleep duration** |  |  |
| Overall Sample | 6.21 (0.09) | 6.93 (0.09) |
| Daily Boost | 6.04 (0.15) | 6.88 (0.15) |
| Bedtime stories | 6.03 (0.22) | 6.63 (0.19) |
| Sleep sounds | 6.30 (0.15) | 7.08 (0.15) |
| Sleep Skills | 6.63 (0.24) | 7.14 (0.23) |
| **PHQ-4** |  |  |
| Overall Sample | 4.57 (0.29) | 3.07 (0.25) |
| Daily Boost | 4.45 (0.49) | 2.91 (0.46) |
| Bedtime stories | 4.79 (0.55) | 3.41 (0.45) |
| Sleep sounds | 4.97 (0.67) | 2.58 (0.46) |
| Sleep Skills | 4.04 (0.60) | 3.60 (0.68) |
| **SWEMWBS** |  |  |
| Overall Sample | 20.48 (0.31) | 22.38 (0.35) |
| Daily Boost | 20.33 (0.48) | 22.62 (0.66) |
| Bedtime stories | 20.52 (0.71) | 21.63 (0.71) |
| Sleep sounds | 20.16 (0.64) | 22.40 (0.57) |
| Sleep Skills | 22.12 (0.78) | 22.70 (0.91) |
| **WPAI** |  |  |
| *Absenteeism* |  |  |
| Overall Sample | 4.04 (0.70) | 2.46 (0.59) |
| Daily Boost | 5.16 (1.33) | 2.22 (0.79) |
| Bedtime stories | 3.47 (1.17) | 2.61 (1.24) |
| Sleep sounds | 4.76 (1.76) | 2.87 (1.49) |
| Sleep Skills | 1.73 (0.91) | 2.21 (1.46) |
| *Presenteeism* |  |  |
| Overall Sample | 42.84 (1.86) | 23.76 (1.87) |
| Daily Boost | 45.42 (3.16) | 22.27 (2.85) |
| Bedtime stories | 44.14 (4.31) | 25.19 (4.04) |
| Sleep sounds | 41.61 (3.85) | 25.33 (4.12) |
| Sleep Skills | 38.08 (3.76) | 22.92 (4.76) |
| *Total work impairment* |  |  |
| Overall Sample | 44.41 (1.93) | 22.98 (1.97) |
| Daily Boost | 47.26 (3.28) | 23.57 (2.97) |
| Bedtime stories | 45.55 (4.46) | 26.42 (4.27) |
| Sleep sounds | 43.34 (4.10) | 26.70 (4.37) |
| Sleep Skills | 39.16 (3.77) | 23.78 (4.94) |
| *Activity impairment* |  |  |
| Overall Sample | 48.68 (1.90) | 26.51 (2.06) |
| Daily Boost | 49.39 (3.27) | 25.00 (3.26) |
| Bedtime stories | 47.93 (3.79) | 30.00 (4.47) |
| Sleep sounds | 49.38 (4.40) | 30.32 (4.65) |
| Sleep Skills | 47.31 (3.94) | 20.80 (4.58) |
| **PSA Cognitive** |  |  |
| Overall Sample | 24.32 (0.60) | 18.91 (0.60) |
| Daily Boost | 26.08 (1.01) | 18.67 (0.97) |
| Bedtime stories | 24.41 (1.05) | 20.63 (1.20) |
| Sleep sounds | 23.19 (1.40) | 17.77 (1.37) |
| Sleep Skills | 22.31 (1.26) | 18.92 (1.42) |
| **PSA Somatic** |  |  |
| Overall Sample | 14.52 (0.50) | 12.33 (0.40) |
| Daily Boost | 14.80 (0.84) | 11.80 (0.59) |
| Bedtime stories | 14.72 (1.13) | 12.96 (0.92) |
| Sleep sounds | 15.28 (1.14) | 12.42 (0.79) |
| Sleep Skills | 12.85 (0.89) | 12.52 (1.10) |
| **CERQ Adaptive** |  |  |
| Overall Sample | 5.97 (0.12) | 6.33 (0.14) |
| Daily Boost | 5.97 (0.21) | 6.57 (0.24) |
| Bedtime stories | 5.81 (0.29) | 6.10 (0.31) |
| Sleep sounds | 5.91 (0.23) | 6.09 (0.26) |
| Sleep Skills | 6.22 (0.25) | 6.42 (0.30) |
| **CERQ Maladaptive** |  |  |
| Overall Sample | 5.35 (0.12) | 5.26 (0.12) |
| Daily Boost | 5.30 (0.23) | 5.06 (0.23) |
| Bedtime stories | 5.53 (0.23) | 5.27 (0.20) |
| Sleep sounds | 5.33 (0.26) | 5.46 (0.25) |
| Sleep Skills | 5.30 (0.25) | 5.36 (0.22) |

SE = standard error of the estimated marginal means (EMM) from the ITT sample; t0 = baseline, t1 = post-intervention (4 weeks). Abbreviations: SD-SF = PROMIS Sleep Disturbance Short Form; SRI-SF = PROMIS Sleep-Related Impairment Short Form; PHQ-4 = Patient Health Questionnaire; SWEMWBS = Short Warwick-Edinburgh Mental Wellbeing Scale; WPAI = Work Productivity and Activity Impairment; PSA = Pre-Sleep Arousal; CERQ = Cognitive Emotion Regulation Questionnaire.

**Table S4.** Estimated marginal means (PP Sample) from linear mixed effect models with standardised effect sizes.

| **Outcome** | **Estimates (mean, SE)** | | | **P value**  **(Within-group)** | **P value**  **(Between-group)** | **Hedges g [95% CI]**  **(Between-group)** |
| --- | --- | --- | --- | --- | --- | --- |
|  | **t0** | **t1** | **t0 - t1** |  |  |  |
| **SD-SF** |  |  |  |  |  |  |
| Daily Boost | 59.6 (1.06) | 49.3 (1.09) | 10.34 (1.19) | <.001 |  |  |
| Bedtime Stories | 55.7 (1.38) | 50.5 (1.42) | 5.22 (1.55) | 0.001 | 0.010* | -0.61 [-1.07, -0.15] |
| Sleep Sounds | 58.0 (1.32) | 48.7 (1.33) | 9.25 (1.46) | <.001 | 0.566 | -0.13 [-0.57, 0.31] |
| Sleep Skills | 55.8 (1.32) | 47.7 (1.48) | 8.06 (1.62) | <.001 | 0.260 | -0.27 [-0.73, 0.19] |
| Sleep Tools | 56.6 (0.80) | 49.0 (0.81) | 7.57 (0.89) | <.001 | 0.067 | -0.33 [-0.67, 0.01] |
| **SRI-SF** |  |  |  |  |  |  |
| Daily Boost | 61.00 (1.23) | 51.2 (1.25) | 9.78 (1.16) | <.001 |  |  |
| Bedtime Stories | 60.07 (1.60) | 53.0 (1.63) | 7.71 (1.51) | <.001 | 0.278 | -0.26 [-0.71, 0.20] |
| Sleep Sounds | 61.00 (1.52) | 51.9 (1.63) | 9.10 (1.42) | <.001 | 0.711 | -0.08 [-0.52, 0.36] |
| Sleep Skills | 59.8 (1.68) | 50.0 (1.71) | 9.83 (1.58) | <.001 | 0.980 | 0.01 [-0.45, 0.47] |
| Sleep Tools | 60.5 (0.92) | 51.7 (0.93) | 8.87 (0.86) | <.001 | 0.527 | -0.11 [-0.45, 0.23] |
| **Sleep Duration*** |  |  |  |  |  |  |
| Daily Boost | 6.04 (0.15) | 6.89 (0.15) | -0.85 (0.13) | <.001 |  |  |
| Bedtime Stories | 6.03 (0.19) | 6.58 (0.20) | -0.55 (0.18) | 0.002 | 0.181 | 0.32 [-0.14, 0.77] |
| Sleep Sounds | 6.30 (0.18) | 7.08 (0.18) | -0.79 (0.16) | <.001 | 0.789 | 0.06 [-0.38, 0.50] |
| Sleep Skills | 6.63 (0.20) | 7.19 (0.21) | -0.55 (0.18) | 0.003 | 0.196 | 0.31 [-0.15, 0.78] |
| Sleep Tools | 6.31 (0.11) | 6.95 (0.11) | -0.64 (0.10) | <.001 | 0.222 | 0.22 [-0.12, 0.56] |
| **PHQ4** |  |  |  |  |  |  |
| Daily Boost | 4.45 (0.45) | 2.98 (0.46) | 1.47 (0.39) | <.001 |  |  |
| Bedtime Stories | 4.79 (0.58) | 3.25 (0.60) | 1.54 (0.51) | 0.003 | 0.915 | 0.03 [-0.42, 0.47] |
| Sleep Sounds | 4.97 (0.56) | 2.56 (0.56) | 2.41 (0.48) | <.001 | 0.134 | 0.34 [-0.10, 0.79] |
| Sleep Skills | 4.04 (0.62) | 3.60 (0.62) | 0.44 (0.54) | 0.413 | 0.122 | -0.38 [-0.84, 0.08] |
| Sleep Tools | 4.63 (0.34) | 3.10 (0.34) | 1.53 (0.30) | <.001 | 0.908 | 0.02 [-0.32, 0.36] |
| **PHQ4 - Depression** | | | | | | |
| Daily Boost | 1.98 (0.23) | 1.27 (0.24) | 0.71 (0.21) | 0.001 |  |  |
| Bedtime Stories | 2.10 (0.30) | 1.39 (0.31) | 0.72 (0.28) | 0.010 | 0.989 | 0.00 [-0.45, 0.45] |
| Sleep Sounds | 2.25 (0.29) | 1.22 (0.29) | 1.03 (0.26) | <.001 | 0.344 | 0.22 [-0.23, 0.66] |
| Sleep Skills | 1.65 (0.32) | 1.35 (0.33) | 0.30 (0.29) | 0.297 | 0.249 | -0.28 [-0.74, 0.18] |
| Sleep Tools | 2.02 (0.18) | 1.31 (0.18) | 0.71 (0.16) | <.001 | 0.985 | 0.00 [-0.34, 0.34] |
| **PHQ4 - Anxiety** |  |  |  |  |  |  |
| Daily Boost | 2.47 (0.26) | 1.71 (0.27) | 0.76 (0.23) | 0.002 |  |  |
| Bedtime Stories | 2.69 (0.34) | 1.87 (0.35) | 0.82 (0.31) | 0.008 | 0.881 | 0.04 [-0.41, 0.48] |
| Sleep Sounds | 2.72 (0.32) | 1.34 (0.33) | 1.38 (0.29) | <.001 | 0.097 | 0.38 [-0.07, 0.82] |
| Sleep Skills | 2.38 (0.36) | 2.25 (0.36) | 0.14 (0.32) | 0.663 | 0.118 | -0.38 [-0.84, 0.08] |
| Sleep Tools | 2.61 (0.20) | 1.79 (0.20) | 0.82 (0.18) | <.001 | 0.840 | 0.04 [-0.30, 0.37] |
| **SWEMWBS*** |  |  |  |  |  |  |
| Daily Boost | 20.3 (0.55) | 22.6 (0.57) | -2.33 (0.52) | <.001 |  |  |
| Bedtime Stories | 20.5 (0.72) | 22.0 (0.74) | -1.44 (0.68) | 0.037 | 0.306 | 0.24 [-0.21, 0.69] |
| Sleep Sounds | 20.2 (0.68) | 22.5 (0.69) | -2.33 (0.64) | <.001 | 0.993 | 0.00 [-0.44, 0.44] |
| Sleep Skills | 21.1 (0.76) | 22.7 (0.77) | -1.59 (0.71) | 0.027 | 0.407 | 0.20 [-0.26, 0.66] |
| Sleep Tools | 20.6 (0.41) | 22.4 (0.42) | -1.82 (0.39) | <.001 | 0.437 | 0.14 [-0.20, 0.48] |
| **WPAI**  **Absenteeism** | | | | | | |
| Daily Boost | 5.16 (1.07) | 2.48 (1.11) | 2.69 (1.18) | 0.024 |  |  |
| Bedtime Stories | 3.47 (1.38) | 2.50 (1.42) | 0.97 (1.51) | 0.521 | 0.371 | -0.21 [-0.66, 0.24] |
| Sleep Sounds | 4.76 (1.34) | 2.80 (1.36) | 1.95 (1.44) | 0.176 | 0.697 | - 0.09 [-0.53, 0.35] |
| Sleep Skills | 1.73 (1.46) | 2.15 (1.51) | -0.42 (1.59) | 0.793 | 0.120 | -0.38 [-0.84, 0.08] |
| Sleep Tools | 3.41 (0.80) | 2.50 (0.82) | 0.91 (0.87) | 0.297 | 0.226 | -0.22 [-0.56, 0.12] |
| **Presenteeism** |  |  |  |  |  |  |
| Daily Boost | 45.4 (3.09) | 22.6 (3.20) | 22.9 (3.37) | <.001 |  |  |
| Bedtime Stories | 44.1 (3.97) | 24.6 (4.09) | 19.6 (4.31) | <.001 | 0.549 | - 0.14 [-0.59, 0.31] |
| Sleep Sounds | 41.6 (3.84) | 25.0 (3.89) | 16.6 (4.11) | <.001 | 0.240 | -0.27 [-0.71, 0.18] |
| Sleep Skills | 38.1 (4.19) | 23.4 (4.33) | 14.7 (4.56) | 0.002 | 0.154 | -0.35 [-0.81, 0.12] |
| Sleep Tools | 41.4 (2.29) | 24.4 (2.35) | 17.0 (2.48) | <.001 | 0.164 | -0.25 [-0.59, 0.09] |
| **Total work impairment** | | | |  |  |  |
| Daily Boost | 47.3 (3.22) | 23.9 (3.33) | 23.3 (3.44) | <.001 |  |  |
| Bedtime Stories | 45.5 (4.14) | 25.7 (4.26) | 19.8 (4.40) | <.001 | 0.533 | - 0.15 [-0.60, 0.30] |
| Sleep Sounds | 43.3 (4.01) | 26.3 (4.06) | 17.0 (4.19) | <.001 | 0.246 | -0.26 [-0.71, 0.18] |
| Sleep Skills | 39.2 (3.33) | 24.2 (4.52) | 15.0 (4.66) | 0.002 | 0.151 | -0.35 [-0.81, 0.11] |
| Sleep Tools | 42.8 (2.39) | 25.5 (2.45) | 17.3 (2.53) | <.001 | 0.161 | -0.25 [-0.59, 0.09] |
| **Activity impairment** | | | | | | |
| Daily Boost | 49.4 (3.27) | 25.4 (3.35) | 24.0 (3.47) | <.001 |  |  |
| Bedtime Stories | 47.9 (4.25) | 29.6 (4.37) | 18.4 (4.53) | <.001 | 0.329 | -0.23 [-0.68, 0.22] |
| Sleep Sounds | 49.4 (4.04) | 30.0 (4.10) | 19.3 (4.25) | <.001 | 0.401 | -0.19 [-0.63, 0.25] |
| Sleep Skills | 47.3 (4.49) | 20.7 (4.56) | 26.6 (4.73) | <.001 | 0.649 | 0.11 [-0.35, 0.57] |
| Sleep Tools | 48.3 (2.45) | 27.1 (2.50) | 21.2 (2.59) | <.001 | 0.527 | -0.11 [-0.45, 0.23] |
| **PSA - Cognitive** |  |  |  |  |  |  |
| Daily Boost | 26.1 (0.98) | 18.7 (1.01) | 7.34 (1.02) | <.001 |  |  |
| Bedtime Stories | 24.4 (1.27) | 20.7 (1.31) | 3.75 (1.34) | 0.006 | 0.035* | -0.50 [-0.96, -0.04] |
| Sleep Sounds | 23.2 (1.21) | 17.7 (1.23) | 5.51 (1.25) | <.001 | 0.260 | -0.26 [-0.70, 0.19] |
| Sleep Skills | 22.3 (1.35) | 18.9 (1.37) | 3.45 (1.39) | 0.015 | 0.026* | -0.54 [-1.01, -0.08] |
| Sleep Tools | 23.3 (0.74) | 19.0 (0.75) | 4.32 (0.76) | <.001 | 0.020* | -0.42 [-0.77, -0.08] |
| **PSA - Somatic** |  |  |  |  |  |  |
| Daily Boost | 14.8 (0.75) | 11.9 (0.77) | 2.88 (0.72) | <.001 |  |  |
| Bedtime Stories | 14.7 (0.98) | 12.8 (1.00) | 1.88 (0.93) | 0.046 | 0.392 | -0.20 [-0.65, 0.25] |
| Sleep Sounds | 15.3 (0.93) | 12.3 (0.94) | 2.98 (0.87) | <.001 | 0.935 | 0.02 [- 0.42, 0.46] |
| Sleep Skills | 12.8 (1.03) | 12.6 (1.05) | 0.30 (0.97) | 0.758 | 0.034* | -0.52 [-0.98, -0.05] |
| Sleep Tools | 14.4 (0.75) | 12.6 (0.57) | 1.81 (0.54) | <.001 | 0.235 | -0.21 [-0.55, 0.13] |
| **CERQ – Adaptive*** | | | | | | |
| Daily Boost | 5.97 (0.21) | 6.58 (0.21) | - 0.61 (0.17) | <.001 |  |  |
| Bedtime Stories | 5.81 (0.27) | 6.13 (0.28) | - 0.32 (0.22) | 0.148 | 0.287 | 0.25 [-0.20, 0.70] |
| Sleep Sounds | 5.91 (0.26) | 6.11 (0.26) | - 0.20 (0.21) | 0.320 | 0.125 | 0.35 [-0.09, 0.79] |
| Sleep Skills | 6.22 (0.29) | 6.41 (0.29) | - 0.20 (0.23) | 0.395 | 0.142 | 0.36 [-0.10, 0.82] |
| Sleep Tools | 5.97 (0.16) | 6.21 (0.16) | -0.24 (0.12) | 0.056 | 0.074 | 0.32 [-0.02, 0.66] |
| **CERQ - Maladaptive** | | | | | | |
| Daily Boost | 5.30 (0.20) | 5.06 (0.20) | 0.24 (0.16) | 0.148 |  |  |
| Bedtime Stories | 5.53 (0.26) | 5.24 (0.26) | 0.28 (0.21) | 0.181 | 0.857 | 0.04 [- 0.41, 0.49] |
| Sleep Sounds | 5.33 (0.25) | 5.44 (0.25) | -0.11 (0.20) | 0.568 | 0.174 | -0.31 [-0.09, 0.79] |
| Sleep Skills | 5.30 (0.27) | 5.31 (0.28) | - 0.01 (0.22) | 0.955 | 0.365 | -0.22 [-0.68, 0.82] |
| Sleep Tools | 5.39 (0.15) | 5.34 (0.15) | 0.05 (0.12) | 0.670 | 0.350 | -0.17 [-0.51, 0.17] |

SE = standard error of the estimated marginal means (EMM) from the PP analysis (participants that completed at least 12 session); standardised effect sizes are Hedges’ g with 95% confidence intervals (CI); t0 = baseline, t1 = post-intervention (4 weeks). Abbreviations: SD-SF = PROMIS Sleep Disturbance Short Form; SRI-SF = PROMIS Sleep-Related Impairment Short Form; PHQ-4 = Patient Health Questionnaire; SWEMWBS = Short Warwick-Edinburgh Mental Wellbeing Scale; WPAI = Work Productivity and Activity Impairment; PSA = Pre-Sleep Arousal; CERQ = Cognitive Emotion Regulation Questionnaire.

* Variables where higher values reflect positive outcomes; for all other variables, higher values indicate negative outcomes.
